# Transgenerational epimutations induced by multi-generation drought imposition mediate rice plant’s adaptation to drought condition

**DOI:** 10.1038/srep39843

**Published:** 2017-01-04

**Authors:** Xiaoguo Zheng, Liang Chen, Hui Xia, Haibin Wei, Qiaojun Lou, Mingshou Li, Tiemei Li, Lijun Luo

**Affiliations:** 1 Shanghai Agrobiological Gene Center, Shanghai, China; 2 College of Plant Sciences and Technology, Huazhong Agricultural University, Wuhan, China

## Abstract

Epigenetic mechanisms are crucial mediators of appropriate plant reactions to adverse environments, but their involvement in long-term adaptation is less clear. Here, we established two rice epimutation accumulation lines by applying drought conditions to 11 successive generations of two rice varieties. We took advantage of recent technical advances to examine the role of DNA methylation variations on rice adaptation to drought stress. We found that multi-generational drought improved the drought adaptability of offspring in upland fields. At single-base resolution, we discovered non-random appearance of drought-induced epimutations. Moreover, we found that a high proportion of drought-induced epimutations maintained their altered DNA methylation status in advanced generations. In addition, genes related to transgenerational epimutations directly participated in stress-responsive pathways. Analysis based on a cluster of drought-responsive genes revealed that their DNA methylation patterns were affected by multi-generational drought. These results suggested that epigenetic mechanisms play important roles in rice adaptations to upland growth conditions. Epigenetic variations have morphological, physiological and ecological consequences and are heritable across generations, suggesting that epigenetics can be considered an important regulatory mechanism in plant long-term adaptation and evolution under adverse environments.

DNA cytosine methylation is a stable and heritable epigenetic marker with essential roles in the regulation of endogenous gene expression and formation of heterochromatin in plants^1^,^2^,^3^,^4^. Naturally occurring DNA methylation variations (epimutations) in a single gene locus can result in heritable morphological variation without alteration of the underlying DNA sequence of genes, such as *epiD1* and *epiFIE1* in rice^5^,^6^. Previous studies have revealed that heritable epimutations play important roles in plant growth and development^7^. In *Arabidopsis thaliana*, RNA-directed *de novo* DNA methylation of CG dinucleotides is stably inherited, and in offspring, this methylation affects the transcription of the target gene^8^. Moreover, transgenerational epimutations have critical functions in the adaptations of plants to their changing environments because epimutation states can be modified by environmental factors and are reversible^9^,^10^.

Epimutations not only occur spontaneously^5^,^11^,^12^,^13^ but also can be induced by biotic and abiotic stresses in plants^14^,^15^. In addition, many stress-induced epimutations can be faithfully transmitted to progeny^15^. Under benign greenhouse conditions, the number of spontaneous single cytosine epimutations is greater than that of DNA sequence mutations, but spontaneous regional epimutations arise at rates similar to those of spontaneous mutations in *A. thaliana*^11^,^12^,^16^. However, there has been very little work to determine the emergence and accumulation of epimutations using natural conditions, which are much more stressful. Furthermore, the investigation of transgenerational inheritance of stress-induced epimutations and its function in the long-term adaptation of plants is still in its infancy.

Rice is an important model species for the evolutionary study of cereals and other monocotyledonous plants. Although many drought-responsive genes have been identified, epigenetic mechanisms, which could improve plant long-term adaptation to environmental challenges, have received less attention than other mechanisms of drought stress response in rice, and previous studies have been limited by short-term treatment and low resolution^9^,^17^.

To improve our understanding of the roles of epigenetic mechanisms in plant long-term adaptation to adverse natural environments, we established two rice epimutation accumulation (epi-MA) lines through exerting 11 successive generations of drought imposition on a drought avoidance variety, Huhan3 (*O. sativa* L. ssp. *japonica*), and a drought tolerance variety, II-32B (*O. sativa* L. ssp. *indica*), by single-seed descent (Fig. S1). Using whole-genome bisulfite sequencing^18^, we generated highly integrated DNA methylome maps for 32 individuals from these two epi-MA lines. High-resolution analysis allowed an investigation into the frequency and distribution of epimutations induced by multi-generational drought, including differentially methylated positions (DMPs), single-cytosine methylation polymorphisms (SMPs) and differentially methylated regions (DMRs).

## Results

### Improved drought adaptability of advanced generations

**Figure 1:**
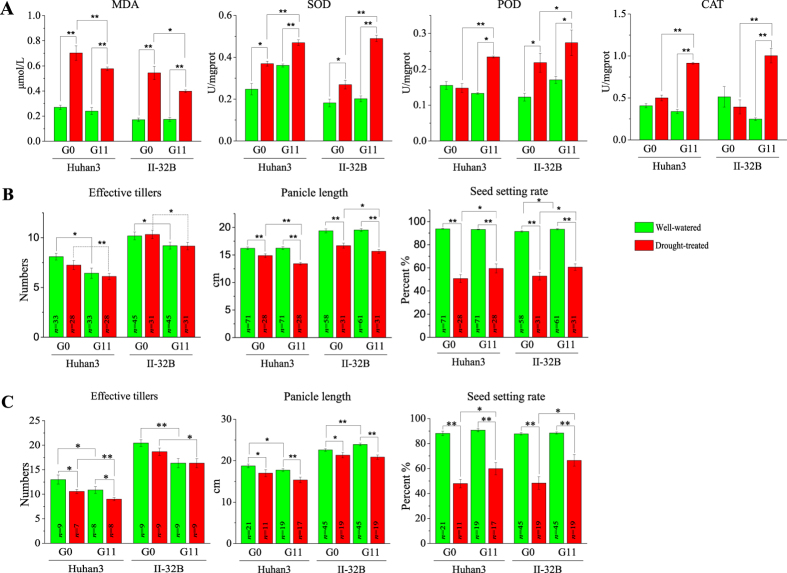
Improved drought adaptability of advanced generations. **(A**) Physiological indexes that indicate the drought tolerance level of rice plants during drought, including the MDA content, which represents oxidative damage to the plant cells, and the activities of SOD, POD and CAT, important components of the oxygen radical-scavenging enzyme system. (**B**) Morphological phenotypes in upland fields. (**C**) Morphological phenotypes in PVC cylinders.

To identify whether the drought resistance level of the offspring improved, we analyzed the physiological and morphological differences between G0 (the original generation) and G11 (the eleventh generation) under stress conditions. After treatment with 20% polyethylene glycol 6000 (osmotic stress) for 48 h, the malondialdehyde (MDA) content, which is a valuable indicator of oxidative damage, was significantly lower in G11 than in G0 in both Huhan3 and II-32B plants (Fig. 1A). In contrast, superoxide dismutase (SOD), peroxidase (POD) and catalase (CAT), three major components of endogenous oxygen radical scavenging enzyme systems, had higher activities in G11 than in G0 (Fig. 1A). Less cell damage and increased reactive oxygen species (ROS) scavenging capacity suggested an improved dehydration tolerance level of advanced generations after multi-generational drought.

The comprehensive drought response of multiple generations was subsequently analyzed in an upland field. Compared to G0 plants, the numbers of effective tillers and the panicle lengths of G11 plants were decreased, while the seed setting rates were increased in both Huhan3 and II-32B plants under drought stress (Fig. 1B). The same results were observed when the plants were cultivated in polyvinylchloride (PVC) cylinders (Fig. 1C and Fig. S1C). These observations indicated that the advanced generations achieve a better balance between “survival strategy” (decreasing the number of effective tillers to reduce water consumption) and “reproductive strategy” (increasing the seed setting rate to maintain the yield) than the original generation. In addition, G11 plants had fewer effective tillers than G0 plants under normal water conditions (Fig. 1B,C), indicating that the phenotypic changes acquired after eleven generations of drought exertion remained stable when the water stress was removed. Taken together, multi-generation drought imposition led to physiological and morphological changes in advanced generations, which enhanced their adaptability to drought conditions, thereby increasing the dehydration tolerance level and producing a better balance between survival and reproduction.

### Directional changes of drought-induced epimutations

Thirty-two methylomes were analyzed, including two rice varieties (Huhan3 and II-32B), four generations per variety (G0; G11; G10, the tenth generation; and G10R1, a recovery generation derived from cultivating G10 under well-watered conditions for one more generation), two treatments per generation (drought-treated and well-watered), and two biological replicates per treatment (sibling plants) (Fig. S1). On average, the strand-specific coverage depths reached approximately 13.45× and 11.21× per individual and covered 84.64% and 76.42% of all cytosine residues present in the reference genomes for Huhan3 and II-32B, respectively (Table S1).

Of the 124,164,895 (Huhan3) and 109,979,307 (II-32B) cytosine residues with sequencing coverage ≥3, on average, 39,919,967 (Huhan3) and 17,827,432 (II-32B) cytosine residues were methylated in each line. We subsequently evaluated 86,672,538 and 76,450,669 cytosine residues that had at least threefold coverage in all 16 individuals from Huhan3 and II-32B, respectively. DMPs, SMPs and DMRs between the drought-treated and untreated plants and among multiple generations were analyzed to investigate the impact of multi-generation drought imposition on DNA methylation patterns of rice plants (Table S2).

**Figure 2:**
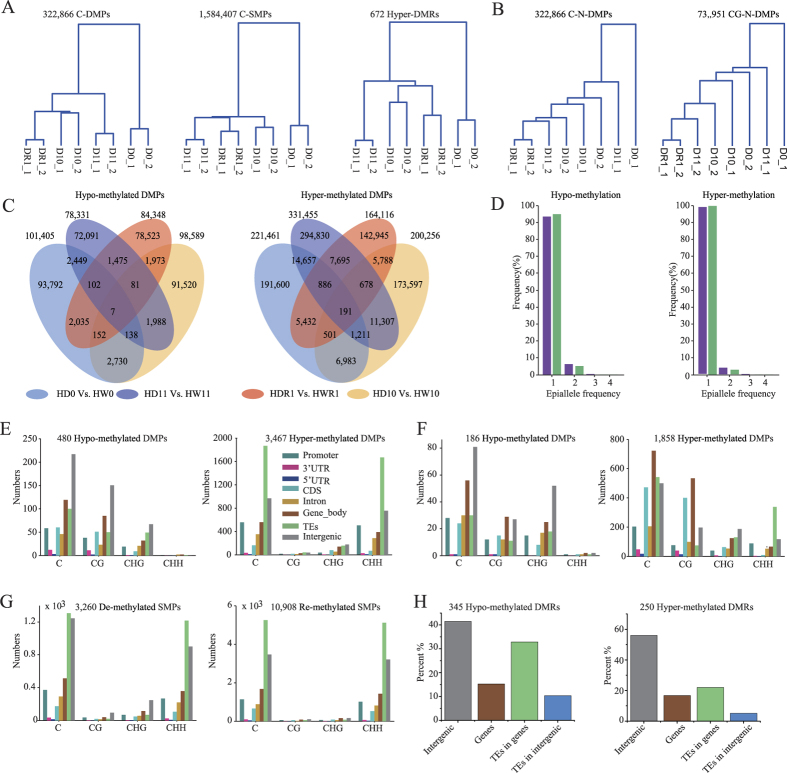
Non-random appearance of drought-induced epimutations. (**A**) Hierarchical clusters according to epimutations that would be induced by drought stress, i.e., epimutations between D0 and W0. (**B**) Hierarchical clusters according to an arbitrary selection of non-epimutation cytosine residues. (**C**) Epimutations between the drought-treated and well-watered Huhan3 plants of four generations in Huhan3. (**D**) The proportions of recurring epimutations with different frequencies. The distribution of drought-induced DMPs whose recurrence frequencies are equal to or greater than three on genic regions in Huhan3 (**E**) and II-32B (**F**). (**G**) Distribution of drought-induced DMRs whose recurrence frequencies are equal to or greater than two times on genic regions in Huhan3.

Epimutations between D0 and W0 were considered to be potentially induced by drought stress. Hierarchical clustering by these epimutations separated the G0 plants and other generations into distinct groups in both the Huhan3 and II-32B varieties, suggesting that long-term drought impacts the DNA methylation patterns of these positions and regions (Fig. 2A and Fig. S2). The same tendencies were apparent in clusters based on CG-, CHG- and CHH-DMPs or SMPs (Fig. S2). However, when non-epimutations were arbitrarily selected between D0 and W0, the clusters became much more random, suggesting that not all cytosine residues changed their methylation statuses after drought (Fig. 2B). Thus, multi-generational drought exerted considerable influence on the DNA methylation statuses of certain positions and regions. Furthermore, the influence was directional because the epimutations in offspring generations were clustered together. Otherwise, the epimutations clustered more randomly.

To further determine whether the effects of long-term drought imposition on epimutations were directional, we considered epimutations between D10 and D0 as accumulated epimutations that were obtained after 10 generations of drought. If the effects were not directional, hierarchical clustering according to these epimutations would be much more random. The clusters grouped siblings together but separated G0 from the other generations, i.e., G11 and G10R1 were close to G10. This result suggested that the effects of long-term drought on epimutations are directional, due to the fact that closer generations exhibited more similar DNA methylation patterns (Fig. S3). Similar results were observed in clusters based on CG-, CHG- and CHH-DMPs/SMPs between D10 and D0.

Although epimutations were directionally affected, their numbers did not increase linearly with time. We found 312,695 accumulated C-DMPs between G0 and G11 in Huhan3 (Table S2), with an average of 18,426 C-DMPs accumulated per generation in expectation. In contrast, we identified 275,374 C-DMPs between D10 and D11, representing epimutations obtained through a single generation of drought imposition, a much larger number than we would have expected. Similar results were observed in the analysis of the DMPs in II-32B plants and in the analysis of SMPs in both varieties, suggesting that many epimutations were not stably inherited during long-term drought.

### Drought-induced epimutations occurred in “hot spots”

To investigate whether the drought-induced epimutations occurred in random positions or in “hot spots,” we analyzed the epimutations between the drought-treated and untreated plants of four generational comparisons in both Huhan3 and II-32B (Fig. S4). If epimutations arose randomly, we would expect less than 1% of events to be recurring. Among the total 349,056 hypo-methylated and 858,301 hyper-methylated DMPs that presented in at least one of the four generational comparisons in Huhan3 plants, we observed 13,130 (3.76%) and 55,329 (6.45%) DMPs that occurred more than once, respectively (Fig. 2C,D). In II-32B, the numbers of recurring events were 6,668 (2.78% of 240,165) and 33,703 (5.79% of 582,427), respectively (Fig. S4A,B). These percentages of recurring DMP events were much higher than the random frequencies that we expected in both varieties, suggesting that drought-induced DMPs occur non-randomly. Because the data analysis did not consider heritable DMPs, which could transmit their changed methylation statuses to their offspring, the actual proportions should be greater than those presented above.

The same analysis was performed for SMPs and DMRs (Fig. S4). In Huhan3, 3.89% (127,723 of 3,284,317) of the de-methylated SMPs and 6.15% (264,962 of 4,305,238) of the re-methylated SMPs were recurring epimutations. In II-32B, the percentages were 3.58% (85,971 of 2,401,056) and 5.76% (173,633 of 3,017,017), respectively. In addition, 5.95% (345 of 5,796) of hypo-methylated DMRs and 7.62% (250 of 3,280) of hyper-methylated DMRs were recurring epimutations in Huhan3. In II-32B, the percentages were 6.40% (262 of 4,093) and 2.76% (55 of 1,995), respectively. As with the DMPs, the frequencies of recurring SMPs and DMRs were much higher than the random frequency, indicating that certain positions and regions are “hot spots” and are particularly prone to hyper-/re-methylation or hypo-/de-methylation in response to drought stress.

Distribution analysis of epimutations whose recurrence frequencies were equal to or greater than 3 times revealed that the hypo-methylated DMPs mostly occurred in CG and CHG contexts, were essentially absent from the CHH context, and were mainly located in the intergenic regions in both varieties (Fig. 2E,F). Hyper-methylated recurring DMPs mostly occurred in the CHH context and were in the transposon elements (TEs) in Huhan3 (Fig. 2E). However, in II-32B, they mostly occurred in CG and CHG contexts and mainly appeared in the gene bodies, especially in the CDS regions (Fig. 2F). For recurring SMPs, both re- and de-methylated SMPs mostly occurred in the CHH context, were essentially absent from the CG and CHG contexts, and mainly appeared in TEs in both varieties (Fig. 2G and Fig. S4G,H). For recurring DMRs, both hyper- and hypo-methylated DMRs mainly localized to the intergenic regions (Fig. 2H and Fig. S4K,L). It was noteworthy that hyper-methylated DMPs and re-methylated SMPs predominated in the recurring events after drought treatment, suggesting that more positions tended to be methylated in response to drought stress. Different distribution features between DMPs and SMPs on the genic regions might mean different functions between them.

### Drought-induced epimutations maintained their changed statuses in advanced generations

Among the DMPs that could be induced by drought stress (i.e., DMPs between D0 and W0), we found that in Huhan3, 54.80% (55,566 of 101,405) of the hypo-methylated DMPs and 74.69% (165,418 of 221,461) of the hyper-methylated DMPs maintained their changed DNA methylation statuses in advanced generations (G10, G11 and G10R1) under drought stress. In II-32B, the percentages were 49.52% (25,697 of 51,889) and 78.74% (116,319 of 147,727), respectively. In total, 68.44% and 71.14% of these drought-induced DMPs maintained their changed DNA methylation statuses in advanced generations under drought stress in Huhan3 and II-32B, respectively. Among drought-induced SMPs, we found that 52.69% (329,730 of 625,822) of de-methylated SMPs and 39.65% (380,102 of 958,585) of re-methylated SMPs maintained their changed DNA methylation statuses in advanced generations in Huhan3. In II-32B, the percentages were 65.51% (332,031 of 506,838) and 37.96% (314,531 of 828,486), respectively. In total, 44.80% and 48.42% of the drought-induced SMPs maintained their changed DNA methylation statuses in advanced generations in Huhan3 and II-32B, respectively. The high percentages of drought-induced epimutations that maintained their changed statuses in advanced generations suggest that these epimutations could be considered epigenetic markers (DMP- or SMP-markers) that participate in the response to drought stress and in the long-term adaptation of plants to drought conditions.

### Transgenerational inheritance of accumulated epimutations

Among accumulated DMPs in Huhan3, 5.59% (7,535 of 134,686) of the hypo-methylated and 13.03% (18,279 of 140,330) of the hyper-methylated DMPs transmitted their changed methylation status to their offspring (G11 and G10R1) (Fig. S5A). In II-32B, the percentages were 5.14% (3,547 of 69,012) and 8.19% (19,666 of 240,242), respectively (Fig. S5B). In total, approximately 9.39% and 7.51% of the accumulated DMPs were transgenerational in Huhan3 and II-32B, among which hyper-methylated DMPs accounted for 70.81% and 84.72%, respectively. Distribution analysis revealed that these transgenerational accumulated DMPs mainly occurred in the CG and CHG contexts (Fig. S5C,D). Interestingly, the CG-DMPs were mainly located in the gene bodies, especially in the CDS regions, rather than in introns. However, CHG- and CHH-DMPs were mainly located in TEs. For accumulated-SMPs, approximately 14.86% and 15.60% were transgenerational in Huhan3 and II-32B, among which de-methylated SMPs accounted for 59.30% and 70.67%, respectively (Fig. S5E,F). In contrast to DMPs, transgenerational SMPs mainly occurred in the CHH context (Fig. S5G,H). Furthermore, de-methylated SMPs were mostly distributed in gene bodies, especially in the CDS regions, while re-methylated SMPs were mostly distributed in TEs.

**Figure 3:**
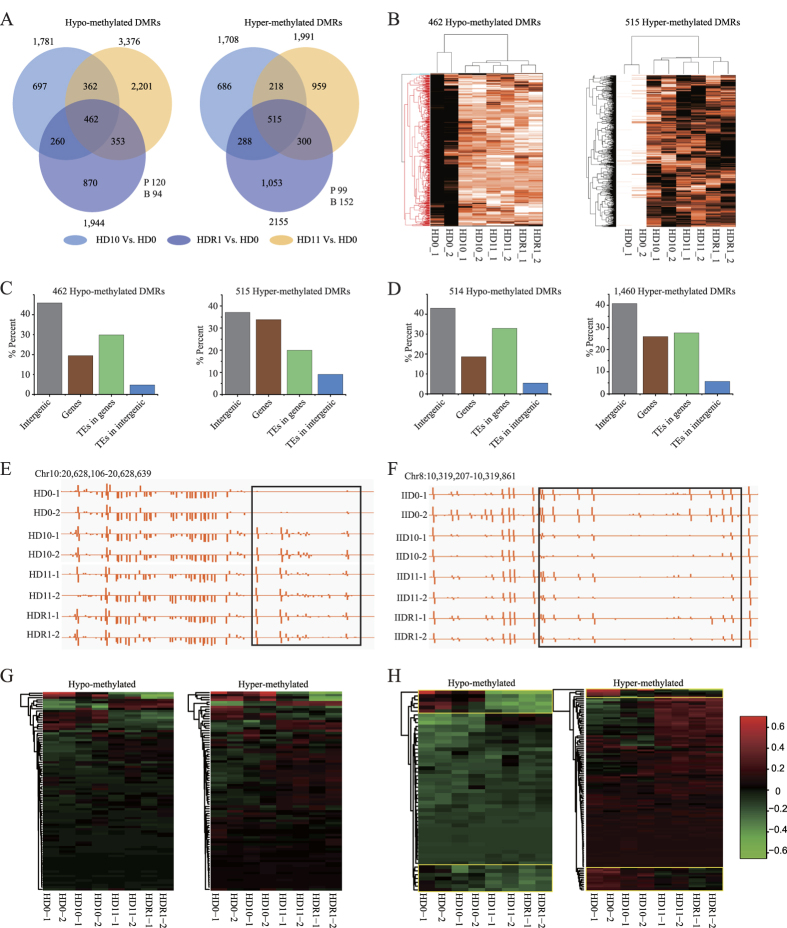
Transgenerational inheritance of accumulated epimutations. (**A**) Transgenerational inheritance of epimutations that accumulated after ten generations of drought imposition. (**B**) Heatmap according to the DNA methylation levels of transgenerational epimutations in multiple generations of Huhan3. (**C**) Distribution of transgenerational DMRs in Huhan3. (**D**) Distribution of transgenerational DMRs in II-32B. (**E**) A hypermethylated transgenerational DMR in Huhan3. (**F**) A hypomethylated transgenerational DMR in Huhan3. (**G**) Expression heatmap of genes whose promoter regions overlapped with transgenerational DMRs in Huhan3. (**H**) Expression heatmap of genes whose bodies overlapped with transgenerational DMRs in Huhan3.

More importantly, 25.94% (462 of 1,781) of the hypo-methylated and 30.15% (515 of 1,708) of the hyper-methylated accumulated DMRs were transgenerational in Huhan3 (Fig. 3A,B). In II-32B, the percentages were 35.87% (514 of 1,433) and 22.00% (1,460 of 6,636), respectively (Fig. S5I). In total, 28.00% and 24.46% of the accumulated-DMRs were transgenerational in Huhan3 and II-32B, respectively. Hypo-methylated DMRs mainly distributed in TEs, which overlapped with gene bodies, in addition to intergenic regions, in both varieties (Fig. 3C,D). Large numbers of the accumulated epimutations could transmit their changed methylation status to their offspring in at least one drought-treated generation (from G10 to G11) and one well-watered generation (from G10 to G10R1), suggesting important roles of these epimutations in passing stress-treatment “memory” and in plant long-term adaptation. Interestingly, most of these transgenerational DMPs and DMRs were hyper-methylated, while most of the transgenerational SMPs were de-methylated.

### Epimutation-related genes directly participate in stress-responsive pathways

To identify the roles of drought-induced epimutations in the stress response, the functions of non-TE genes that were related to both recurring and transgenerational epimutations were analyzed. GO (Gene Ontology) analysis indicated that the products of these non-TE genes are involved in a wide range of functions in rice plants, including signal transduction, flower development, pollination, responses to biotic and abiotic stress, and reproduction. These pathways are associated with plant development and the stress response. We subsequently determined that 14.04–19.19% of these non-TE genes directly participate in stress-responsive pathways (Table S3). For example, *LOC_Os08g33720*, a gene that contained 12 hypo-methylated CG-DMPs with recurrence frequencies ≥3 in Huhan3, encodes a putative lactate/malate dehydrogenase. Its product is a component of the mitochondrion and has binding and catalytic activities. This gene participates in the response to abiotic stimuli. The same analyses were performed for the non-TE genes that are related to transgenerational accumulated epimutations (Table S4). Among these non-TE genes, 15.28%–26.47% of them directly participate in stress responsive pathways. For example, *LOC_Os04g42784* contained 29 transgenerational de-methylated SMPs in II-32B; the locus encodes a DNA mismatch repair protein and participates in the response to abiotic stimuli.

A single DMP or SMP would have limited contribution to gene expression and phenotypic modification. We then analyzed non-TE genes that were related to DMRs that were DMP-clustered regions. In this study, 31.01–40.08% of the drought-induced recurring DMRs and transgenerational accumulated DMRs overlapped with the promoter regions or bodies of functional genes (Table S5). GO analysis revealed that 5.26–19.09% of these non-TE genes directly participate in stress responses. For example, in Huhan3, *LOC_Os10g38720*, a gene encoding a putative glutathione S-transferase, overlapped with a transgenerational hyper-methylated DMR (Chr10:20628250-20628549) in its promoter region (Fig. 3E). This gene participates in the response to abiotic stimuli. In II-32B, another gene, *LOC_Os08g16860*, which encodes the OsFBX282 - F-box domain containing protein, overlapped with a transgenerational hypo-methylated DMR (Chr8:10319550-10319899) in its gene body (Fig. 3F).

To investigate whether the expression levels of genes related to transgenerational DMRs change after multi-generation drought exertion, we performed mRNA sequencing for eight drought-treated samples of Huhan3 (Fig. 3G,H and Table S6). Transgenerational DMR-associated genes were divided into two groups: one group was composed of genes whose promoter regions overlap with transgenerational DMRs, and another group was composed of genes whose bodies overlap with transgenerational DMRs. An expression heat map based on the 112 hypo- and 114 hyper-methylated genes of the first group revealed no significant correlations between the DNA methylation statuses of promoter DMRs and gene expression levels (Fig. 3G). However, a heat map based on the 77 hypo- and 137 hyper-methylated genes of the second group revealed that genes with hyper-methylated DMRs in their bodies tend to be up-regulated (Fig. 3H). More importantly, 16.88% (13 of 77) of the hypo- and 12.40% (17 of 137) of the hyper-methylated genes maintained their changed expression patterns in offspring, suggesting that the gene expression variations show transgenerational inheritance (Fig. 3H).

### CG contexts in the CDS regions of drought-responsive genes tend to be hyper-methylated

**Figure 4:**
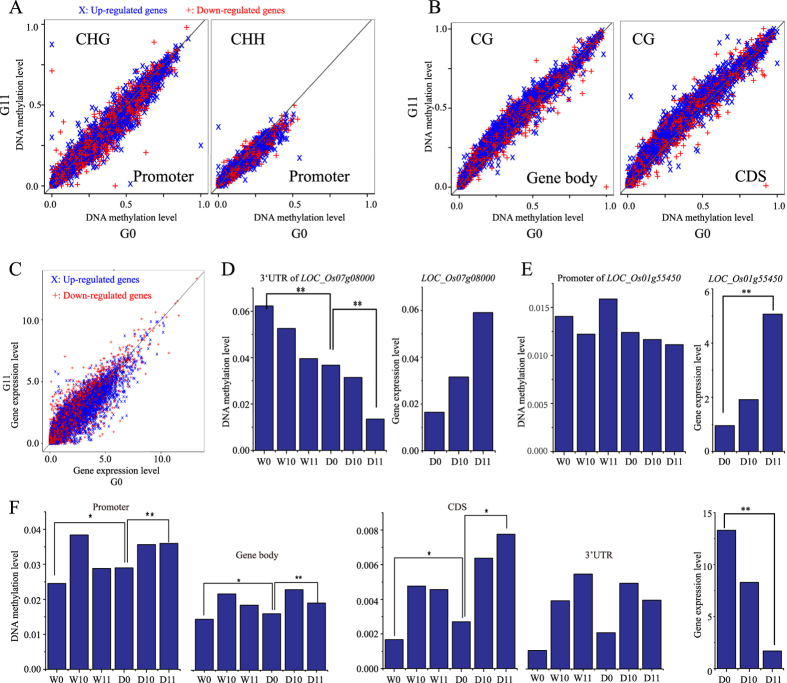
DNA methylation variations between G0 and G11 in drought-responsive genes. (**A**) Non-CG methylation variations in promoter regions. (**B**) CG methylation variations in gene bodies and CDS regions. (**C**) Expression variations between G0 and G11 in up- and down-regulated drought-responsive genes. (**D**) The DNA methylation level of the 3′UTR of *LOC_Os07g08000* decreased after multi-generational drought imposition in Huhan3. However, the expression of this gene increased in G11 compared to G0. (**E**) The DNA methylation level of the *LOC_Os01g55450* promoter region decreased after multi-generational drought in Huhan3. However, its expression significantly increased in G11. (**F**) The promoter region, gene body, CDS and 3′UTR of *LOC_Os03g12290* were hyper-methylated after multi-generational drought. Its expression level was significantly decreased in G11 under drought conditions.

A total of 9,113 rice drought-responsive genes that changed their expression patterns after drought treatment were collected from 7 published experiments (Table S7). We subsequently analyzed variations in DNA methylation patterns in the promoter regions, gene bodies, CDS regions, 5′ untranslated regions (5′UTRs) and 3′UTRs of these drought-responsive genes between G0 and G11. We found that non-CG contexts in promoter regions tend to be hypo-methylated in G11 (Fig. 4A and Figs S6 and S7). However, the CG contexts in gene bodies, especially in CDS regions, tend to be hyper-methylated (Fig. 4B). Furthermore, non-CG methylation was essentially absent from the CDS regions (Figs S6 and S7). In addition, the number of down-regulated drought-responsive genes tended to be more highly expressed in G11 than in G0 under drought stress (Fig. 4C). Thus, multi-generational drought exerted considerable influence on both DNA methylation and the gene expression patterns of drought-responsive genes.

Drought-induced phenotypic modulations, such as altered tiller numbers and seed setting rates, are complex quantitative traits and are regulated by a number of genes. The enrichment effects of slight expression changes that are mediated by epigenetic variations of these genes could lead to significant phenotypic modulation. Therefore, from the Q-TARO (QTL Annotation Rice Online) database, we collected 763 gene loci that have been mapped and associate with the phenotypic modulations in our study. We then analyzed the variations in DNA methylation levels of each genic region (promoter region, gene body, 5′UTR, 3′UTR, intron and CDS) of these genes between treated and untreated plants and among multiple generations. The results revealed that 18 genes tended to be hypo-methylated under drought conditions and after multi-generational drought (i.e., their DNA methylation levels followed the pattern W0 > D0 > D11) in Huhan3. For example, *LOC_Os07g08000*, a gene related to sterility, would associate with the seed setting rate. The DNA methylation level of its 3′UTR tended to be hypo-methylated, and its expression level was higher in G11 than in G0 (Fig. 4D, Fig. S9). The promoter region of *LOC_Os01g55450*, a gene related to drought resistance, tended to be hypo-methylated, and its expression level was significantly increased in G11 compared to G0 (Fig. 4E, Fig. S9). Furthermore, there were 582 phenotype-associated genes that tended to be hyper-methylated under drought conditions and after drought stress (i.e., their DNA methylation levels followed the pattern W0 < D0 < D11) in Huhan3. For example, *LOC_Os03g12290*, a gene associated with tiller numbers, drought resistance and source activity, had hyper-methylated regions in the promoter, gene body, CDS and 3′UTR under drought conditions and after drought stress and was accompanied by significantly decreased gene expression (Fig. 4F). In addition, 230 and 116 of these phenotype-associated genes tended to be hypo-methylated and hyper-methylated in II-32B, respectively. It is extremely likely that variations in DNA methylation patterns and in the expression levels of genes that associate with phenotypic modulation after multi-generational drought play important roles in rice adaptation to upland ecosystems.

### Huhan3 and II-32B display different DNA methylation patterns and variations

**Figure 5:**
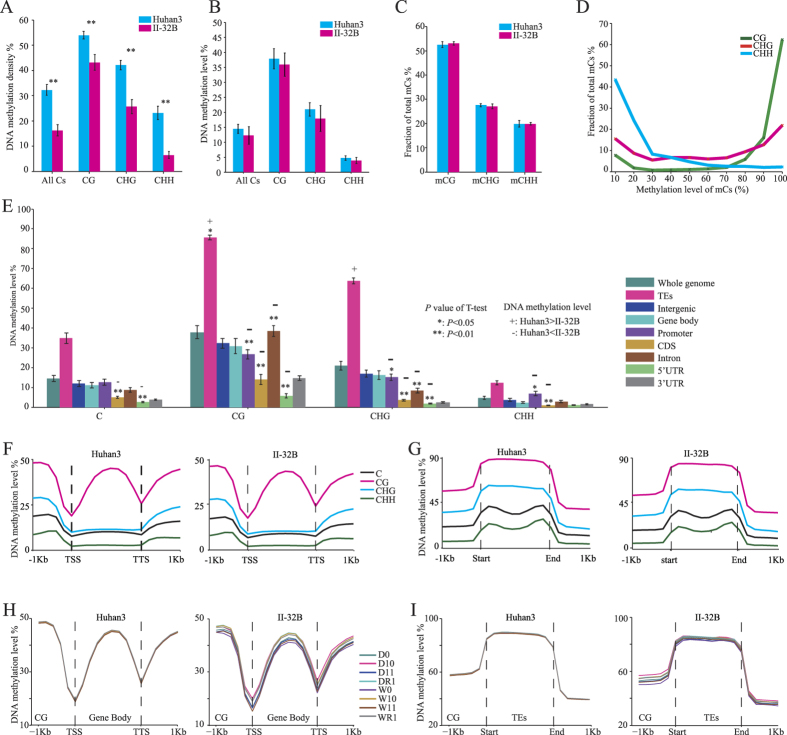
DNA methylation patterns of both rice varieties. (**A**) DNA methylation densities for all contexts. (**B**) DNA methylation levels for all contexts. (**C**) The relative proportions of mCs in three sequence contexts. (**D**) Distribution of methylation level for mCs in each sequence context. (**E**) DNA methylation levels in each sequence context for different genomic regions. Significant differences between Huhan3 and II-32B were determined using *t*-tests. (**F**) Distribution of DNA methylation levels in the gene bodies and 1-kb flanking sequences. (**G**) Distribution of methylation levels in TEs and 1-kb flanking sequences. (**H**) Distribution of CG DNA methylation levels in gene bodies of each strain. (**I**) Distribution of CG DNA methylation levels in TEs of each strain.

To investigate the general DNA methylation pattern of rice and the DNA methylation divergence between the two rice ecotypes, we analyzed the DNA methylation patterns of both Huhan3 and II-32B. For all three contexts considered, Huhan3 had greater DNA methylation density than II-32B, but there were no differences in the DNA methylation levels (Fig. 5A,B). DNA methylation mainly occurred in the CG context, where it had the highest DNA methylation density and level (Fig. 5A–C). Distribution analysis of the methylation level of mCs in each sequence context revealed that mCG tended to be hyper-methylated while mCHH tended to be hypo-methylated (Fig. 5D). In both varieties and all three contexts, within the genomic regions, there was greater methylation of the TEs than other parts (Fig. 5E). It should be noted that Huhan3 had significantly lower DNA methylation levels than II-32B in many genomic regions, including the promoter, intron, exon, 5′UTR, and 3′ UTR, but not the TEs. The CDS regions significantly differed in DNA methylation levels between the two varieties in all contexts.

We further analyzed the distribution of DNA methylation in the gene bodies, TEs and their 1-kb flanking sequences on both sides. As shown in Fig. 5F, in rice genes, the CG methylation level sharply declined from approximately 600 bp upstream of the transcriptional start site (TSS), reached the lowest level at the TSS, and then gradually increased along the gene body. The same pattern occurred at the 3′ ends of the genes, which suggested that a lack of CG methylation around the transcription termination site (TTS) might be as important for gene expression as TSS methylation. Non-CG methylation was essentially absent from the gene bodies, whereas methylation in all contexts was abundant in TEs (Fig. 5G).

In addition, we analyzed the variation in the DNA methylation patterns of gene bodies and TEs in multiple generations. CG methylation had much greater stability than non-CG methylation in both the gene bodies and TEs (Fig. S8). Furthermore, the DNA methylation in Huhan3 plants had less variation than that in II-32B plants, implying that Huhan3 plants had more stable methylation patterns in the gene bodies and TEs than II-32B when subjected to drought stress (Fig. 5H,I and Fig. S8).

## Discussion

Epigenetic mechanisms provide a biological link between plant phenotypes and their exposure to environmental conditions. However, the extent to which epigenetic variation contributes to phenotypes remains to be determined^19^. Here, we found that multi-generational drought exerted considerable influence on the drought adaptation and methylomes of rice plants. After the selective pressure of relatively long-term adverse environments, the offspring generations achieved a better balance between “survival strategy” and “reproductive strategy” than the original generations, which manifested as decreased numbers of effective tillers and increased seed setting rates. In addition, both the average biological yield and the thousand-kernel weight were unchanged between G0 and G11 (Fig. S10A,B). Interestingly, the kernel lengths were decreased, while the kernel widths were increased after drought exertion, indicating that long-term drought changed the seed shapes from elongated ellipses to a more circular shape (Fig. S10C,D). The underlying mechanisms for these changes are unclear.

Stress-induced epimutations arose non-randomly and occurred in “hot spots”, consistent with previous studies showing that drought induces site-specific DNA methylation in rice and that these DNA methylation loci associate with drought tolerance^9^. Moreover, we found that the number of epimutations changed directionally and was transgenerational, consistent with the directional natural selection of modern evolutionary theory. The purpose of their appearance was to modulate plant gene expression to adapt to changing environments. In addition, many of the related genes directly participated in stress-responsive pathways, and the DNA methylation patterns of drought-responsive genes and phenotype-associated genes were affected by multi-generational drought, suggesting that epigenetic mechanisms play important roles in rice long-term adaptation and evolution in the upland ecosystem. Previous studies had determined that genome-wide DNA methylation changes could be caused by a range of environmental stresses and that the inheritance of stress-induced epigenetic variation over generations plays important roles in plant adaption and evolution^20^,^21^,^22^,^23^. For instance, nitrogen deficiency-induced transgenerational DNA methylation changes in rice, which enhance stress tolerance, could be passed on to the subsequent generations^24^. Additionally, heat treatment-induced epigenetic memory in the parental and F1 generations of *A. thaliana* greatly improved the fitness of plants exposed to heat in a later generation (F3)^25^. In allopolyploid orchids, stable epigenetic effects impacted their adaption to the ecological environment^26^. The inheritance of accumulated epimutations showed epigenetic inheritance, which was not due to underlying genetic variation (genetic inheritance)^27^, as all analyses were based on samples from a common ancestor and genetic variation was almost completely absent. In a similar study in *A. thaliana*, RNA-directed *de novo* DNA methylation in CG dinucleotides was stably transmitted to offspring for at least three generations^8^.

Drought stress was performed in upland field, a much more variable and stressful natural environment than the benign greenhouse, and was close to the evolutionary environment of upland rice. Therefore, further study is required to determine the roles of epigenetic mechanisms in differentiation between upland and lowland rice ecotypes and whether the transgenerational epimutations in our study reemerged between them.

High-throughput analysis and single-base resolution allowed the investigation of epimutations in all contexts (CG, CHG and CHH) and in all forms (DMPs, SMPs and DMRs); previous studies have not considered this level of detail due to low resolution^9^,^28^. With respect to the genomic structures in Huhan3 and II-32B, the genome-wide methylation patterns were similar to other rice varieties^29^,^30^. The greater instability of CHH-methylation, rather than CG- and CHG-methylation, was likely due to its maintenance mechanism (Table S2, Fig. S4G,H and Fig. S5G,H). CHH methylation must be regenerated *de novo* by DRM2, and drought stress might affect the reprogramming process. Two rice varieties displayed differences in DNA methylation patterns and in epigenetic variation after drought stress (Fig. 5 and Fig. S8). The drought-resistant variety, Huhan3, had significantly higher methylation density in all contexts but lower methylation levels in many genic regions, except for TEs, than the drought-sensitive variety, II-32B (Fig. 5E).

Epimutations did not distribute randomly throughout the genome^11^. DMPs, SMPs and DMRs were three different epigenetic markers with different distribution features on genic regions (Figs S4 and S5). DMPs mainly occurred in the CG and CHG contexts and were mostly distributed in the intergenic regions, while SMPs mainly occurred in the CHH context and were mostly distributed in TEs. The different distribution features possibly implied different functions between DMPs and SMPs in the stress response; however, most of the previous high-resolution studies focused on only one type of marker^11^,^12^,^31^. Loci with different methylation statuses, when investigated in low-resolution studies using enzyme digestion methods, such as methylation-sensitive amplified polymorphism (MSAP), were determined to be SMPs, and the extent of DNA methylation could not be distinguished^9^,^28^. Therefore, SMPs and DMPs should be discriminated in future studies. In addition, re-methylation events (SMPs) were predominant in both the drought-susceptible and drought-resistant genotypes under drought conditions^28^. Furthermore, 28.68–33.18% of the de-methylated accumulated SMPs were transgenerational, consistent with a previous study that found that 29% of drought-induced DNA methylation changes maintained their statuses after recovery to the normal condition^9^.

TEs account for at least 35% of the total genome in rice^32^. Although usually suppressed by DNA methylation, TEs contributed to the activation of the plant responses to abiotic stress^33^. In *A.thaliana*, DNA demethylases played significant roles in regulating the expression of stress-responsive genes through targeting TEs in their promoter regions^34^. Therefore, DNA methylation variation in promoter TEs could be considered another type of epimutation.

## Materials and Methods

### Plant growth and drought treatment of rice plants

Seeds from a single plant of Huhan3 and II-32B were designated as the original generation (G0). Fifteen of the seeds were grown in an upland field (Hainan, China), and the plants were then exposed to drought stress (soil water content maintained at 30–45% of the maximum field capacity) from the tillering to grain-filling stages, when drought has the largest impact on plant development and can cause severe yield loss. The seeds harvested from the drought-treated plants were considered the first generation (G1). The same procedure was performed for the subsequent ten generations to obtain the G11 seeds. In addition, another 15 seeds of G10 were cultivated under well-watered conditions for one more generation to obtain a recovery generation, G10R1. During 11 successive generations of drought, both well-watered and drought-treated plants were cultivated in glass houses to avoid the effect of raining.

In this study, 32 individual plants were separately cultivated in PVC cylinders with heights of 100 cm and diameters of 20 cm (Fig. S1C). The PVC cylinders were filled with a mixture of silver sand and fine-grained soil, according to a mass ratio of 3:5. The well-watered group experienced irrigation, while the drought-treated group had the water drained off through drainage holes when the plants reached the tillering stage. Leaf samples were collected carefully when the relative water content of the drought-treated leaves reached less than 70%.

### Identification of physiological phenotype

The physiological phenotype of all samples were determined using four-leaf stage seedling plants which cultivated in a climate chamber (CONVIRON CMP6050) to exclude the effects of exogenous environmental factors and to show more consistent phenotype of each generation. In the climate chamber, the temperature was set from 21 °C to 29 °C, continuous light (200 mmol/m^2^/s) was set from 8:00 AM to 22:00 PM (14 h), and the humidity was set from 75% to 80%. The seedlings were cultivated for four weeks with sufficient nutrient solution. Osmotic stress treatment was performed by 20% PEG6000 solution cultivation. 3 seedlings served as an experimental replicate and 3 replicates were used in this study. The content of malondialdehyde (MDA) and the activities of superoxide dismutase (SOD), peroxidase (POD) and catalase (CAT) in plant tissues were determined according to the kit instructions of Nanjing Jiancheng Bioengineering Institute (Nanjing, China).

### BS-Seq libraries construction and sequencing

Genomic DNA was isolated from leaf samples using the Plant Genomic DNA Purification Kit (Qiagen, Germany) and fragmented into 100–300 bp sections by sonication with a Diagenome sonicator. Residual DNA was eliminated with a DNase I treatment, followed by the 3′-end addition of dA and adaptor ligation according to the manufacturer’s instruction (Illumina). The bisulfite conversion of rice DNA was carried out using the ZYMO EZ DNA Methylation-Gold kit (Zymo Research, Irvine, CA, USA). After desalting and size selection, the bisulfite-treated DNAs were PCR amplified with 18 cycles. We subsequently performed a sample quality test and used these qualified DNA samples to construct the BS library. All the sequences were obtained using an Illumina HiSeq 2000. Bisulfite-converted libraries were sequenced with 2× 90-bp paired-end (PE90) reads. The spiked-in lambda phage was used to determine the bisulfite conversation rate.

### Mapping and processing of BS-Seq reads

The raw paired end reads were trimmed and quality control was performed by SeqPrep (https://github.com/jstjohn/SeqPrep) and Sickle (https://github.com/najoshi/sickle) with default parameters. After filtering the low-quality data, the remaining high quality sequences were aligned against the Nipponbare genome, which had high a quality reference genome sequence and gene annotation information, using the mapping tool Bismark (Version 0.10.1) that supports the alignment of bisulfite converted reads^35^. Reads mapped to the same start position for both ends were regarded as clonal duplicates, which might have been generated during the PCR process, and only one of them was kept. The uniquely mapped data were used to obtain methylation information of cytosine for the whole genome as in a previous study^36^. The reference genome sequences, annotations of genes, transposable elements, and other genomic elements studied in our analyses were obtained from ftp://ftp.plantbiology.msu.edu/pub/data/Eukaryotic_Projects/o_sativa/annotation_dbs/pseudomolecules/version_7.0/.

We estimated the bisulfite conversion rate by using a spiked-in unmethylated lambda phage. Methylated cytosines were identified as Becker *et al*.^11^. We conducted binomial tests using the non-conversion rate and T-C sequencing error rate with false positive rate below 5% to exclude mCs that may be the result of non-conversion of cytosine or sequencing errors.

### Methylation level and methylation density analysis

The methylation level of each cytosine was defined as the proportion of reads showing mC among all reads covering the same cytosine. The methylation level of a region was defined as the average methylation level of all Cs in this region. The methylation density was defined as the proportion of mCs among all Cs in the corresponding sequences.

### Identification of DMPs

DMPs represented positions that had differences in the DNA methylation level between samples, i.e. being high methylation in one sample compared to low methylation in another. It was described as “hyper-methylated” or “hypo-methylated”. Only cytosines that were present in all 16 samples per variety and with high-quality sequencing (coverage ≥ 3) were considered for further DMPs analysis. We identified 86,672,538 and 76,450,669 of these cytosines in Huhan3 and II-32B, respectively. Fisher’s exact test (*p* < 0.01) was used to identify the cytosines that significant differences in their methylation level. *p* values from individual tests per site were combined into single P values via conservative Bonferroni correction. Genome-wide false discovery rates (FDRs) were limited to less than 5%. DMPs between the two biological replicates were filtered out when identified DMPs between generations or between treatments with pairwise Fisher’s exact tests.

### Identification of SMPs

SMPs represented positions that had differences in their DNA methylation status between samples, i.e. being methylated in one sample while being unmethylated in another. It was described as “re-methylated” or “de-methylated”. Similar to the identification of DMPs, only cytosines that presented in all 16 samples per variety and with high-quality sequencing (coverage ≥ 3) were considered for further SMPs analysis. Only positions that were the same in the two biological replicates of each sample (i.e. both were either methylated or unmethylated) were considered in the SMPs identification between generations or between treatments. In addition, positions that had the same methylation status in all 16 samples per variety were removed from the SMPs analysis, as they had no variation between generation and between treatments.

### Identification of DMRs

As DMPs clustered regions, it was described as “hyper-methylated” or “hypo-methylated” as well. A 200-bp sliding-window with 50-bp step-size approach was used to screen the DMRs between samples. The DNA methylation levels of different samples were compared pairwise using Fisher’s exact test and the *p* values were adjusted for multiple comparisons using the Benjamini-Hochberg method. Only windows that had adjusted *p* values < 0.01, changed over 1.5-fold in the methylation level, and that contained at least seven DMPs were retained. Finally, neighboring DMRs were combined if the gap between them was less than or equal to 100 bp.

### mRNA-seq libraries construction and sequencing

The total RNA was extracted using a RNAprep pure Plant Kit (Tiangen, China) according to the manufacturer’s instructions, and the genomic DNA was removed using DNase I. The RNA quality and quantity were determined using a 2100 Bioanalyser (Agilent) and ND-2000 (NanoDrop Technologies) respectively. Then 5 μg of high-quality RNA (OD260/280 = 1.8~2.2, OD260/230 ≥ 2.0, RIN ≥ 6.5, 28 S:18 S ≥ 1.0) was used to construct the sequencing library.

The RNA-seq libraries were prepared according to the manufacturer’s instruction from the TruSeq Stranded Total RNA preparation Kit with Ribo-Zero Plant (Illumina, CA). cDNA synthesis, end repair, A-base addition, and ligation of the Illumina-indexed adaptors were performed according to Illumina’s protocol. Libraries were then size selected for cDNA target fragments of 200–300 bp on a 2% Low Range Ultra Agarose gel, followed by PCR amplification using Phusion DNA polymerase (NEB) for 15 cycles. After quantification by TBS380, the paired-end libraries were sequenced with 2 × 100-bp (PE100) reads on an Illumina HiSeq 2500 instrument.

### MSRE-qPCR to confirm WGBS data

400 ng genomic DNA (add 20 pg unmethylated λDNA, Promega) were equally divided into two groups: one was digested with excessive *Hpa* II (5 U, New England Biolabs) for 8 hours in a 37 °C-thermostatic water bath, and another was set as control group (without enzyme digestion). Then, the digested and undigested products were diluted 5~fold with ddH_2_O, respectively. qPCR was performed in a 20 μl volume, containing 4 μl diluted digested or undigested products, 10 μl 2 × SYBR Premix Ex TaqTM (Takara Biotechnology), 0.6 μl forward primers (10 μM), 0.6 μl reverse primers (10 μM), 0.4 μl ROX Reference Dye II (50×) and 4.4 μl ddH_2_O. Three technological replicates were included in our study. Primers for the 3′UTR of *LOC_Os07g08000* (chr07: 4054252…4054523): forward 5′-TGTTTGGCGAGAGAAGCTCC-3′, reverse 5′-AGAATACGGAGTAAATTCATTC-3′. The product was 105 bp, and it contained one CCGG sequence. Primers for the first exon of *LOC_Os01g55450* (chr01: 31946933…31948491): forward 5′-GATGGCATCGGGGACGC-3′, reverse 5′-GTCGTGTGTGCGTGTGTC-3′. The product was 112 bp, and it contained one CCGG sequence. Primers for λDNA: forward 5′-GTAAGGCGTGGGATGTGCTC-3′, reverse 5′-CCGACCGTCGGATAAGACG-3′. The product was 107 bp, and it contained one CCGG sequence. The qPCR program was 95 °C for 30 s; then 40 cycles at 95 °C for 5 s, and 60 °C for 34 s, with an additional dissociation stage in a ABI 7500 thermal cycler. qPCR result of λDNA of digested group was used to confirm sufficient digestion. The DNA methylation level of target CCGG was calculated as 2^−ΔΔCt^*100%, ΔΔCt = Ct (digested product)-Ct (undigested product).

### RT-qPCR to confirm mRNA-sequencing data

cDNA from mRNA-seq libraries construction process were used for RT-qPCR. Primers for *LOC_Os07g08000*: forward 5′-CTCTCCTCGAATCGACAGAATC-3′, reverse: 5′-GTCAGTGATGGATGGTGGATAG-3′. Primers for *LOC_Os01g55450*: forward 5′-AGAGATCATTGAGAGCGATTGG-3′, reverse 5′-CGTCATCAGACAGGTTGTAGAG-3′. RT-qPCR was performed in a 20 μl volume, containing 0.4 μl cDNA, 10 μl 2 × SYBR Premix Ex Taq^TM^ (Takara Biotechnology), 0.6 μl forward primers (10 μM), 0.6 μl reverse primers (10 μM), 0.4 μl ROX Reference Dye II (50×) and 8 μl ddH_2_O. The program was as MSRE-qPCR. The qPCR data were normalized to the expression of the housekeeping *Actin* gene in rice, actin-forward: 5′-AATGAGTAACCACGCTCCGTCA-3′, actin-reverse: 5′-TGCTATGTACGTCGCCATCCAG-3′, and after normalization, the data were presented as fold change relative to the 1 point.

### Collection of rice drought responsive genes

We collected 9,113 rice drought responsive genes which changed their expression patterns after drought treatment in at least 1 of 7 experimental array-combinations that published in GEO (Gene Expression Omnibus) database of NCBI (National Center for Biotechnology Information) and in ArrayExpress database of EMBL-EBI (The European Bioinformatics Institute of the European Molecular Biology Laboratory).

## Additional Information

**Accession Codes**: All whole genome bisulfite sequencing and mRNA-seq files are available from the NCBI 8 Gene Bank database (accession code PRJNA319450).

**How to cite this article**: Zheng, X. *et al*. Transgenerational epimutations induced by multi-generation drought imposition mediate rice plant’s adaptation to drought condition. *Sci. Rep.* **7**, 39843; doi: 10.1038/srep39843 (2017).

**Publisher's note:** Springer Nature remains neutral with regard to jurisdictional claims in published maps and institutional affiliations.

## Supplementary Material

Supplementary Information
