# Supplementary material for: Transgenerational epimutations induced by multi-generation drought imposition mediate rice plant’s adaptation to drought condition
Source: Sci Rep. 2017 Jan 4;7:39843. doi: 10.1038/srep39843 (PMC5209664; doi:10.1038/srep39843)
Supplement: Supplementary Information [file srep39843-s1.pdf]

Transgenerational epimutations induced by multi-generation drought imposition mediate rice plant's adaptation to drought condition

Xiaoguo Zheng[1,2,†], Liang Chen[1,†], Hui Xia[1], Haibin Wei[1], Qiaojun Lou[1], Mingshou Li[1], Tiemei Li[1] and Lijun Luo[1,2*]

[1] Shanghai Agrobiological Gene Center, Shanghai, China

[2] College of Plant Sciences and Technology, Huazhong Agricultural University, Wuhan, China

*Corresponding author E-mail: lijun@sagc.org.cn.

† These authors contributed equally to this work.

Supplementary Figure S1. Scheme of samples used in this study. (A) Original generations (G0s) of both Huhan3 and II-32B were propagated by single-seed descent for 11 generations, with drought exposure from tillering stage to seed filling stage, which is when drought has the largest impact on yield under natural conditions. In addition, the G10 generation was cultivated under well-watered conditions for one more generation to obtain a recovery generation, G10R1. (B) Analysis was based on two biological replicates (sibling plants) of each sample. (C) PVC cylinders used in this study.

Supplementary Figure S2. Hierarchical clusters according to epimutations between D0 and W0 in all contexts. Clusters according to DMPs (A), SMPs (C) and DMRs (E) in Huhan3, and according to DMPs (B), SMPs (D) and DMRs (F) in II-32B.

Supplementary Figure S3. Hierarchical clusters according to epimutations between D10 and W0 in all contexts. Clusters according to DMPs (A), SMPs (C) and DMRs (E) in Huhan3, and according to DMPs (B), SMPs (D) and DMRs (F) in II-32B.

Supplementary Figure S4. **Epimutations between well-watered and drought-treated plants.** (A) Drought-induced DMPs in Huhan3. (B) Drought-induced DMPs in II-32B. (C) Distribution of DMPs whose recurrent frequencies ≥3 on genic regions in Huhan3. (D) Distribution of DMPs whose recurrent frequencies ≥3 on genic regions in II-32B. (E) Drought-induced SMPs in Huhan3. (F) Drought-induced SMPs in II-32B. (G) Distribution of SMPs whose recurrent frequencies ≥3 on genic regions in Huhan3. (H) Distribution of SMPs whose recurrent frequencies ≥3 on genic regions in II-32B. (I) Drought-induced DMRs in Huhan3. (J) Drought-induced DMRs in II-32B. (K) Distribution of recurrent DMRs on genic regions in Huhan3. (L) Distribution of recurrent DMRs on genic regions in II-32B.

Supplementary Figure S5. Transgenerational inheritance of accumulated epimutations. (A) Transgenerational accumulated DMPs in Huhan3. (B) Transgenerational accumulated DMPs in II-32B. (C) Distribution of transgenerational accumulated DMPs on genic regions in Huhan3. (D) Distribution of transgenerational accumulated DMPs on genic regions in II-32B. (E) Transgenerational accumulated SMPs in Huhan3. (F) Transgenerational accumulated SMPs in II-32B. (G) Distribution of transgenerational accumulated SMPs on genic regions in Huhan3. (H) Distribution of transgenerational accumulated SMPs on genic regions in II-32B. (I) Transgenerational accumulated DMRs in II-32B. (J) Heatmap according to DNA methylation level of transgenerational accumulated DMRs in different generations of II-32B.

Supplementary Figure S6. DNA methylation variations between G0 and G11 in all contexts in promoter regions, 5'UTR, gene bodies, CDS and 3'UTR of drought responsive genes of Huhan3.

Supplementary Figure S7. DNA methylation variations between G0 and G11 in all contexts in promoter regions, 5'UTR, gene bodies, CDS and 3'UTR of drought responsive genes of II-32B.

Supplementary Figure S8. DNA methylation patterns of gene bodies and TEs with their 1 Kb flanking sequences. Gene bodies and TEs were divided equally into ten pieces, and their 1-Kb flanking sequences on both sides were divided equally into five pieces. Distribution of DNA methylation level in gene bodies of each strain of Huhan3 (A) and of II-32B (B). Distribution of DNA methylation level in TEs of each strain of Huhan3 (C) and of II-32B (D).

Supplementary Figure S9. Using MSRE-qPCR and RT-qPCR to confirm WGBS data and mRNA-seq data, respectively. (A) DNA methylation level of cytosine at chr7:4054474 from WGBS data. (B) DNA methylation level of cytosine at chr7:4054474 from MSRE-qPCR data. (C)

Relative expression level of *LOC_Os07g08000*. (D) Relative expression level of *LOC_Os01g55450*.

Supplementary Figure S10. Agronomic characters of G0 and G11 under both normal and drought conditions. (A) Biological yield (dry weight of whole plants) per tiller. (B) Thousands kernel weight. (C) Kernel length. (D) Kernel width.

Figure S1

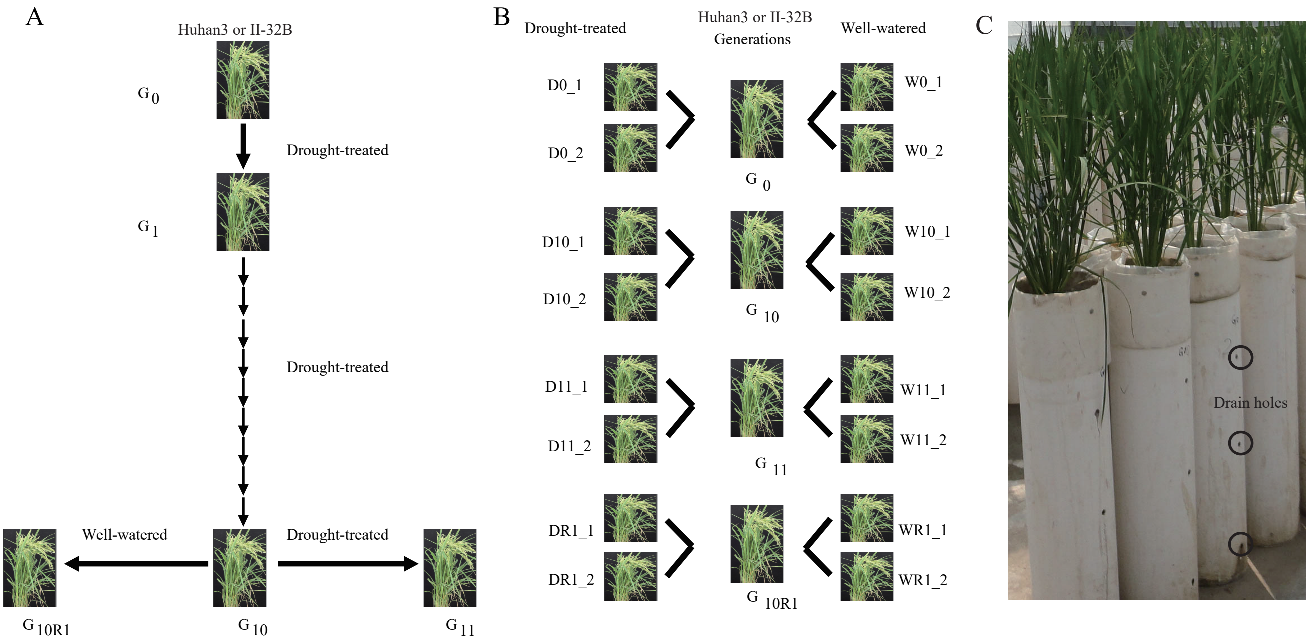
A
Huhan3 or II-32B
G0
Drought-treated
G1
Drought-treated
Well-watered
Drought-treated
G10R1
G10
G11
B
Drought-treated
Huhan3 or II-32B
Generations
Well-watered
D0_1
D0_2
G 0
W0_1
W0_2
D10_1
D10_2
G 10
W10_1
W10_2
D11_1
D11_2
G 11
W11_1
W11_2
DR1_1
DR1_2
G 10R1
WR1_1
WR1_2
C
Drain holes

Figure S2

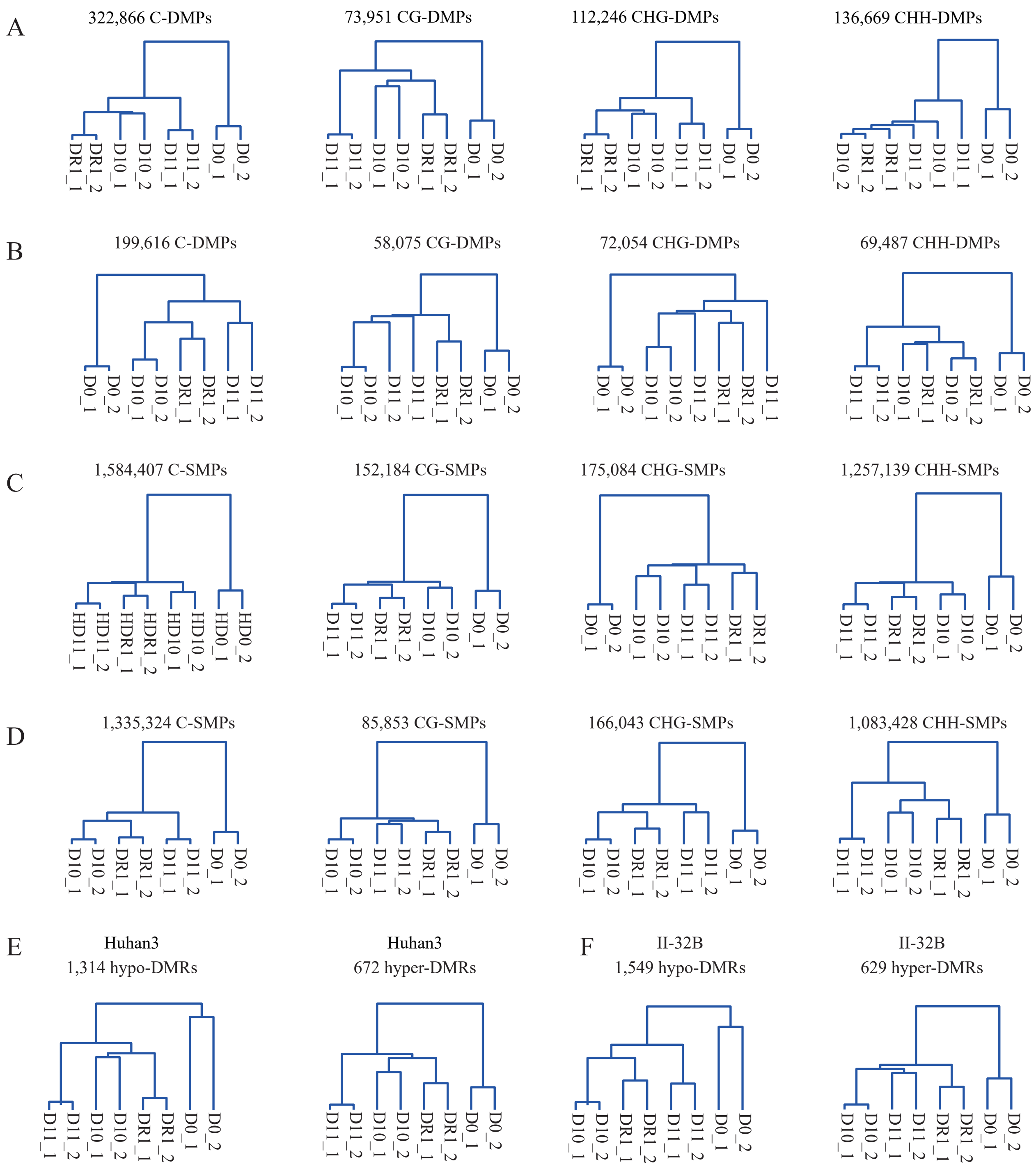
A
322,866 C-DMPs
DR1_1 DR1_2 D10_1 D10_2 D11_1 D11_2 D0_1 D0_2
73,951 CG-DMPs
D11_1 D11_2 D10_1 D10_2 DR1_1 DR1_2 D0_1 D0_2
112,246 CHG-DMPs
DR1_1 DR1_2 D10_1 D10_2 D11_1 D11_2 D0_1 D0_2
136,669 CHH-DMPs
D10_2 DR1_2 DR1_1 D11_2 D10_1 D11_1 D0_1 D0_2
B
199,616 C-DMPs
D0_1 D0_2 D10_1 D10_2 DR1_1 DR1_2 D11_1 D11_2
58,075 CG-DMPs
D10_1 D10_2 D11_2 D11_1 DR1_1 DR1_2 D0_1 D0_2
72,054 CHG-DMPs
D0_1 D0_2 D10_1 D10_2 D11_2 DR1_1 DR1_2 D11_1
69,487 CHH-DMPs
D11_1 D11_2 D10_1 DR1_1 D10_2 DR1_2 D0_1 D0_2
C
1,584,407 C-SMPs
HD11_1 HD11_2 HDR1_1 HDR1_2 HD10_1 HD10_2 HD0_1 HD0_2
152,184 CG-SMPs
D11_1 D11_2 DR1_1 DR1_2 D10_1 D10_2 D0_1 D0_2
175,084 CHG-SMPs
D0_1 D0_2 D10_1 D10_2 D11_1 D11_2 DR1_1 DR1_2
1,257,139 CHH-SMPs
D11_1 D11_2 DR1_1 DR1_2 D10_1 D10_2 D0_1 D0_2
D
1,335,324 C-SMPs
D10_1 D10_2 DR1_1 DR1_2 D11_1 D11_2 D0_1 D0_2
85,853 CG-SMPs
D10_1 D10_2 D11_1 D11_2 DR1_1 DR1_2 D0_1 D0_2
166,043 CHG-SMPs
D10_1 D10_2 DR1_1 DR1_2 D11_1 D11_2 D0_1 D0_2
1,083,428 CHH-SMPs
D11_1 D11_2 D10_1 D10_2 DR1_1 DR1_2 D0_1 D0_2
E
Huhan3
1,314 hypo-DMRs
D11_1 D11_2 D10_1 D10_2 DR1_1 DR1_2 D0_1 D0_2
Huhan3
672 hyper-DMRs
D11_1 D11_2 D10_1 D10_2 DR1_1 DR1_2 D0_1 D0_2
F
II-32B
1,549 hypo-DMRs
D10_1 D10_2 DR1_1 DR1_2 D11_1 D11_2 D0_1 D0_2
II-32B
629 hyper-DMRs
D10_1 D10_2 D11_1 D11_2 DR1_1 DR1_2 D0_1 D0_2

Figure S3

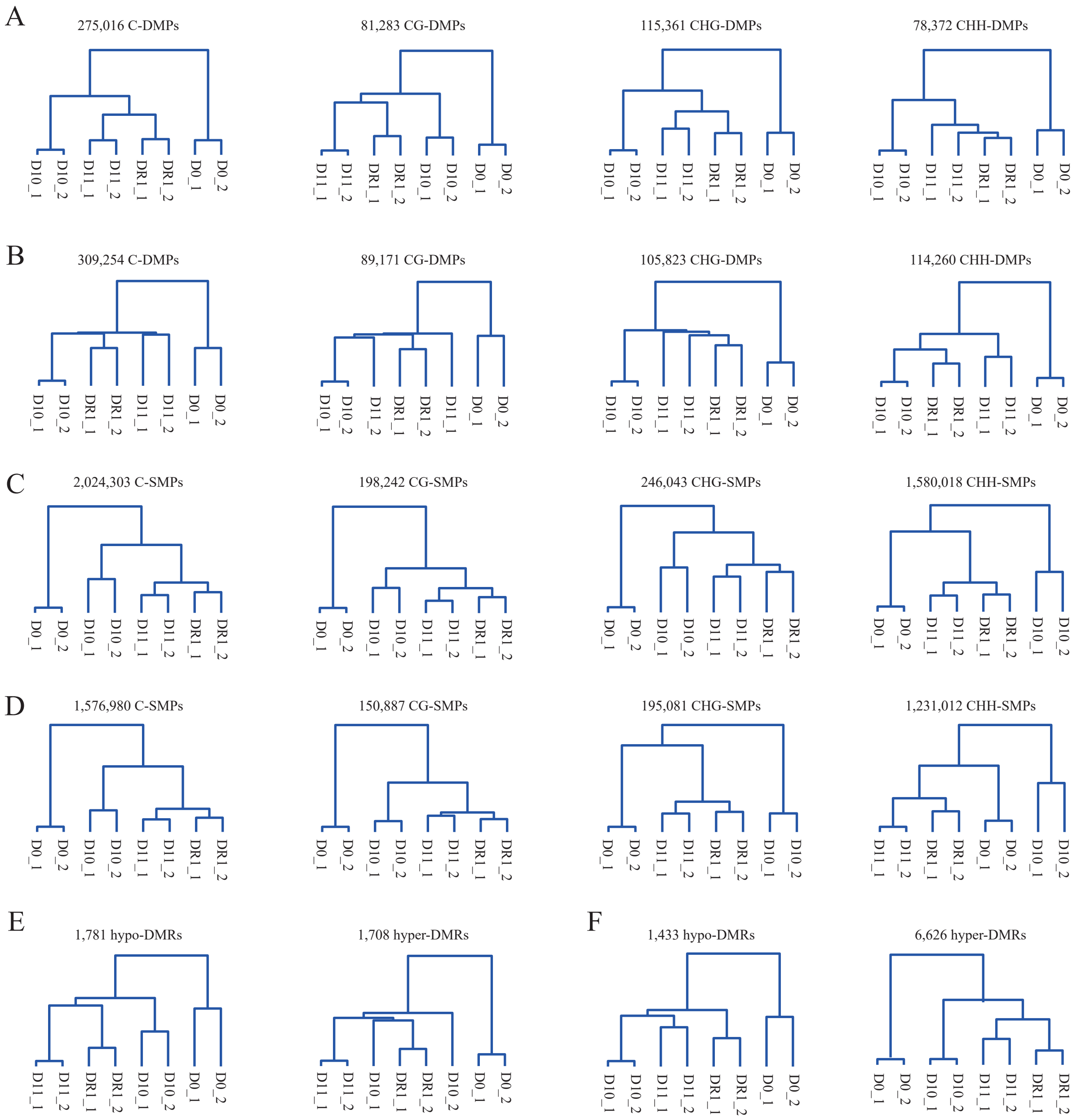
A
275,016 C-DMPs
D10_1 D10_2 D11_1 D11_2 DR1_1 DR1_2 D0_1 D0_2
81,283 CG-DMPs
D11_1 D11_2 DR1_1 DR1_2 D10_1 D10_2 D0_1 D0_2
115,361 CHG-DMPs
D10_1 D10_2 D11_1 D11_2 DR1_1 DR1_2 D0_1 D0_2
78,372 CHH-DMPs
D10_1 D10_2 D11_1 D11_2 DR1_1 DR1_2 D0_1 D0_2
B
309,254 C-DMPs
D10_1 D10_2 DR1_1 DR1_2 D11_1 D11_2 D0_1 D0_2
89,171 CG-DMPs
D10_1 D10_2 D11_2 DR1_1 DR1_2 D11_1 D0_1 D0_2
105,823 CHG-DMPs
D10_1 D10_2 D11_1 D11_2 DR1_1 DR1_2 D0_1 D0_2
114,260 CHH-DMPs
D10_1 D10_2 DR1_1 DR1_2 D11_1 D11_2 D0_1 D0_2
C
2,024,303 C-SMPs
D0_1 D0_2 D10_1 D10_2 D11_1 D11_2 DR1_1 DR1_2
198,242 CG-SMPs
D0_1 D0_2 D10_1 D10_2 D11_1 D11_2 DR1_1 DR1_2
246,043 CHG-SMPs
D0_1 D0_2 D10_1 D10_2 D11_1 D11_2 DR1_1 DR1_2
1,580,018 CHH-SMPs
D0_1 D0_2 D11_1 D11_2 DR1_1 DR1_2 D10_1 D10_2
D
1,576,980 C-SMPs
D0_1 D0_2 D10_1 D10_2 D11_1 D11_2 DR1_1 DR1_2
150,887 CG-SMPs
D0_1 D0_2 D10_1 D10_2 D11_1 D11_2 DR1_1 DR1_2
195,081 CHG-SMPs
D0_1 D0_2 D11_1 D11_2 DR1_1 DR1_2 D10_1 D10_2
1,231,012 CHH-SMPs
D11_1 D11_2 DR1_1 DR1_2 D0_1 D0_2 D10_1 D10_2
E
1,781 hypo-DMRs
D11_1 D11_2 DR1_1 DR1_2 D10_1 D10_2 D0_1 D0_2
1,708 hyper-DMRs
D11_1 D11_2 D10_1 DR1_1 DR1_2 D10_2 D0_1 D0_2
F
1,433 hypo-DMRs
D10_1 D10_2 D11_1 D11_2 DR1_1 DR1_2 D0_1 D0_2
6,626 hyper-DMRs
D0_1 D0_2 D10_1 D10_2 D11_1 D11_2 DR1_1 DR1_2

Figure S4

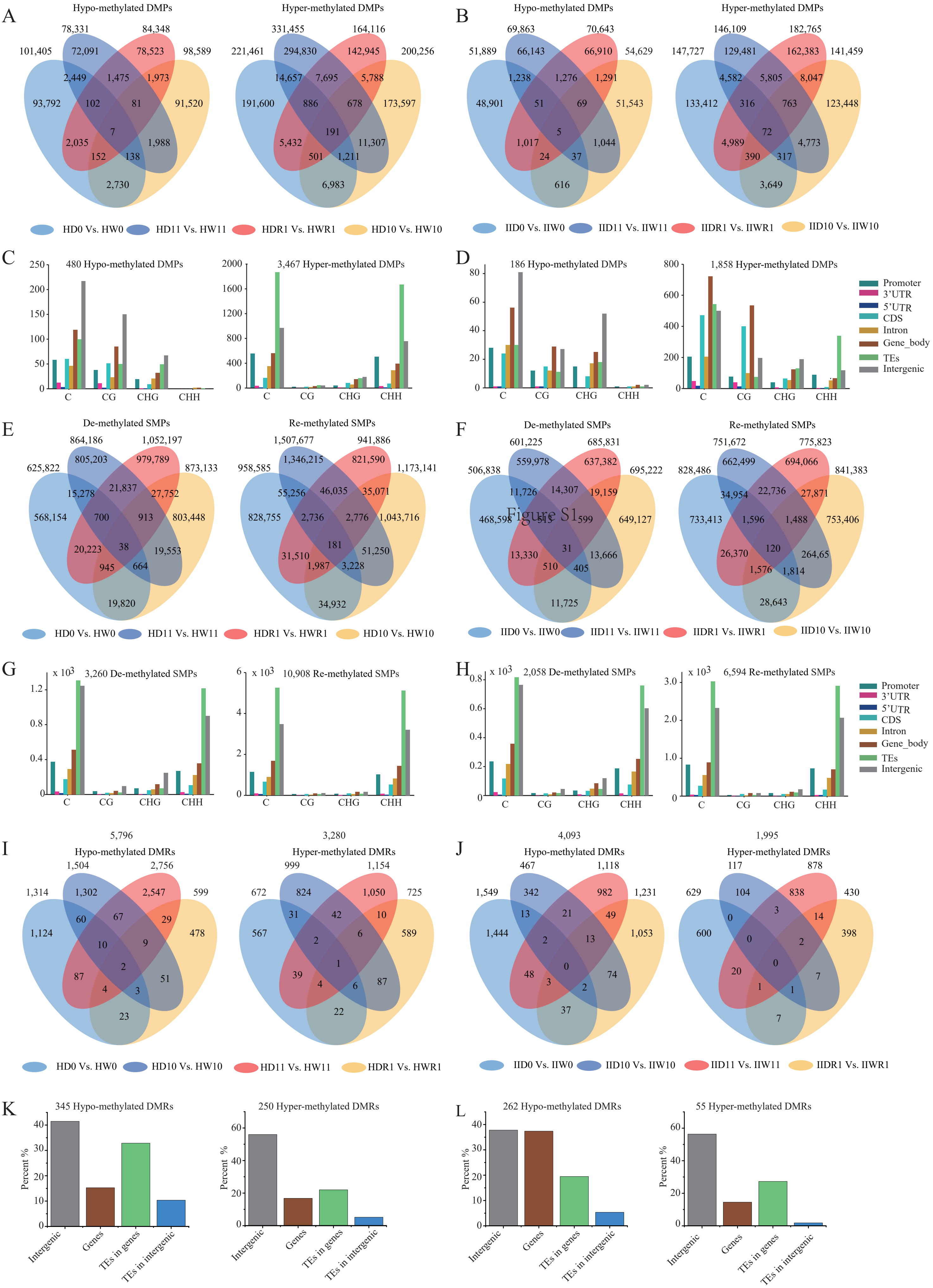
A
Hypo-methylated DMPs
78,331
84,348
101,405
72,091
78,523
98,589
2,449
1,475
1,973
93,792
102
81
91,520
7
2,035
1,988
152
138
2,730
Hyper-methylated DMPs
331,455
164,116
221,461
294,830
142,945
200,256
14,657
7,695
5,788
191,600
886
678
173,597
191
5,432
11,307
501
1,211
6,983
HD0 Vs. HW0
HD11 Vs. HW11
HDR1 Vs. HWR1
HD10 Vs. HW10
B
Hypo-methylated DMPs
69,863
70,643
51,889
66,143
66,910
54,629
1,238
1,276
1,291
48,901
51
69
51,543
5
1,017
1,044
24
37
616
Hyper-methylated DMPs
146,109
182,765
147,727
129,481
162,383
141,459
4,582
5,805
8,047
133,412
316
763
123,448
72
4,989
4,773
390
317
3,649
IID0 Vs. IIW0
IID11 Vs. IIW11
IIDR1 Vs. IIWR1
IID10 Vs. IIW10
C
480 Hypo-methylated DMPs
250
200
150
100
50
0
C
CG
CHG
CHH
3,467 Hyper-methylated DMPs
2000
1600
1200
800
400
0
C
CG
CHG
CHH
D
186 Hypo-methylated DMPs
80
60
40
20
0
C
CG
CHG
CHH
1,858 Hyper-methylated DMPs
800
600
400
200
0
C
CG
CHG
CHH
Promoter
3'UTR
5'UTR
CDS
Intron
Gene_body
TEs
Intergenic
E
De-methylated SMPs
864,186
1,052,197
625,822
805,203
979,789
873,133
15,278
21,837
27,752
568,154
700
913
803,448
38
20,223
19,553
945
664
19,820
Re-methylated SMPs
1,507,677
941,886
958,585
1,346,215
821,590
1,173,141
55,256
46,035
35,071
828,755
2,736
2,776
1,043,716
181
31,510
51,250
1,987
3,228
34,932
HD0 Vs. HW0
HD11 Vs. HW11
HDR1 Vs. HWR1
HD10 Vs. HW10
F
De-methylated SMPs
601,225
685,831
506,838
559,978
637,382
695,222
11,726
14,307
19,159
468,598
515
599
649,127
Figure S1
31
13,330
13,666
510
405
11,725
Re-methylated SMPs
751,672
775,823
828,486
662,499
694,066
841,383
34,954
22,736
27,871
733,413
1,596
1,488
753,406
120
26,370
264,65
1,576
1,814
28,643
IID0 Vs. IIW0
IID11 Vs. IIW11
IIDR1 Vs. IIWR1
IID10 Vs. IIW10
G
x 10^3
3,260 De-methylated SMPs
1.2
0.8
0.4
0
C
CG
CHG
CHH
x 10^3
10,908 Re-methylated SMPs
6
4
2
0
C
CG
CHG
CHH
H
x 10^3
2,058 De-methylated SMPs
0.8
0.6
0.4
0.2
0
C
CG
CHG
CHH
x 10^3
6,594 Re-methylated SMPs
3.0
2.0
1.0
0
C
CG
CHG
CHH
Promoter
3'UTR
5'UTR
CDS
Intron
Gene_body
TEs
Intergenic
I
5,796
Hypo-methylated DMRs
1,504
2,756
1,314
1,302
2,547
599
60
67
29
1,124
10
9
478
2
87
51
4
3
23
3,280
Hyper-methylated DMRs
999
1,154
672
824
1,050
725
31
42
10
567
2
6
589
1
39
87
4
6
22
HD0 Vs. HW0
HD10 Vs. HW10
HD11 Vs. HW11
HDR1 Vs. HWR1
J
4,093
Hypo-methylated DMRs
467
1,118
1,549
342
982
1,231
13
21
49
1,444
2
13
1,053
0
48
74
3
2
37
1,995
Hyper-methylated DMRs
117
878
629
104
838
430
0
3
14
600
0
2
398
0
20
7
1
1
7
IID0 Vs. IIW0
IID10 Vs. IIW10
IID11 Vs. IIW11
IIDR1 Vs. IIWR1
K
345 Hypo-methylated DMRs
Percent %
40
30
20
10
0
Intergenic
Genes
TEs in genes
TEs in intergenic
250 Hyper-methylated DMRs
Percent %
60
40
20
0
Intergenic
Genes
TEs in genes
TEs in intergenic
L
262 Hypo-methylated DMRs
Percent %
40
30
20
10
0
Intergenic
Genes
TEs in genes
TEs in intergenic
55 Hyper-methylated DMRs
Percent %
60
40
20
0
Intergenic
Genes
TEs in genes
TEs in intergenic

Figure S5

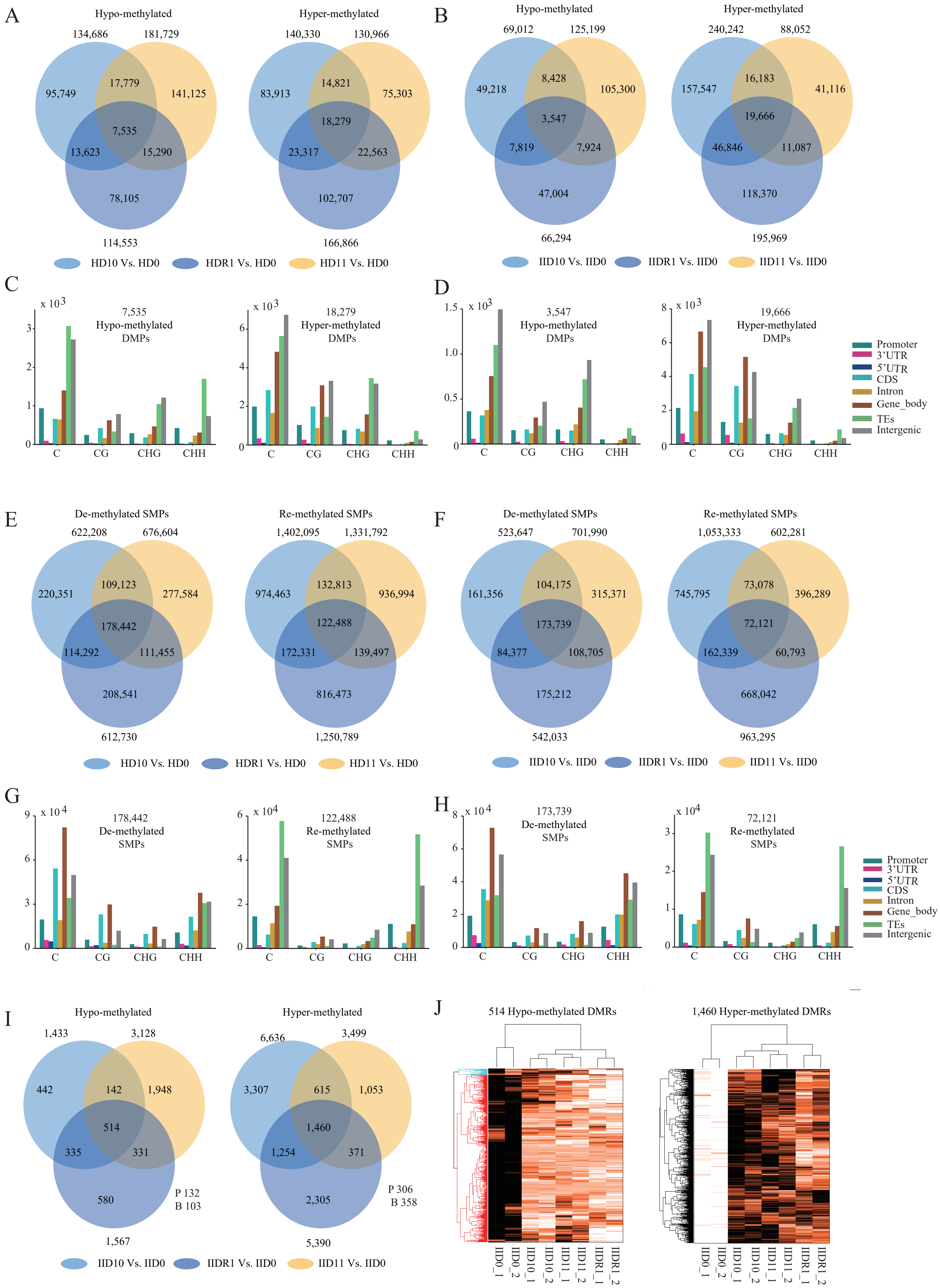
A
Hypo-methylated
134,686
181,729
95,749
17,779
141,125
7,535
13,623
15,290
78,105
114,553
Hyper-methylated
140,330
130,966
83,913
14,821
75,303
18,279
23,317
22,563
102,707
166,866
HD10 Vs. HD0
HDR1 Vs. HD0
HD11 Vs. HD0
B
Hypo-methylated
69,012
125,199
49,218
8,428
105,300
3,547
7,819
7,924
47,004
66,294
Hyper-methylated
240,242
88,052
157,547
16,183
41,116
19,666
46,846
11,087
118,370
195,969
IID10 Vs. IID0
IIDR1 Vs. IID0
IID11 Vs. IID0
C
x 10^3
7,535
Hypo-methylated
DMPs
18,279
Hyper-methylated
DMPs
C
CG
CHG
CHH
D
3,547
Hypo-methylated
DMPs
19,666
Hyper-methylated
DMPs
Promoter
3'UTR
5'UTR
CDS
Intron
Gene_body
TEs
Intergenic
E
De-methylated SMPs
622,208
676,604
220,351
109,123
277,584
178,442
114,292
111,455
208,541
612,730
Re-methylated SMPs
1,402,095
1,331,792
974,463
132,813
936,994
122,488
172,331
139,497
816,473
1,250,789
HD10 Vs. HD0
HDR1 Vs. HD0
HD11 Vs. HD0
F
De-methylated SMPs
523,647
701,990
161,356
104,175
315,371
173,739
84,377
108,705
175,212
542,033
Re-methylated SMPs
1,053,333
602,281
745,795
73,078
396,289
72,121
162,339
60,793
668,042
963,295
IID10 Vs. IID0
IIDR1 Vs. IID0
IID11 Vs. IID0
G
x 10^4
178,442
De-methylated
SMPs
122,488
Re-methylated
SMPs
H
173,739
De-methylated
SMPs
72,121
Re-methylated
SMPs
I
Hypo-methylated
1,433
3,128
442
142
1,948
514
335
331
580
P 132
B 103
1,567
Hyper-methylated
6,636
3,499
3,307
615
1,053
1,460
1,254
371
2,305
P 306
B 358
5,390
IID10 Vs. IID0
IIDR1 Vs. IID0
IID11 Vs. IID0
J
514 Hypo-methylated DMRs
1,460 Hyper-methylated DMRs
IID0_1
IID0_2
IID10_1
IID10_2
IID11_1
IID11_2
IIDR1_1
IIDR1_2

Figure S6

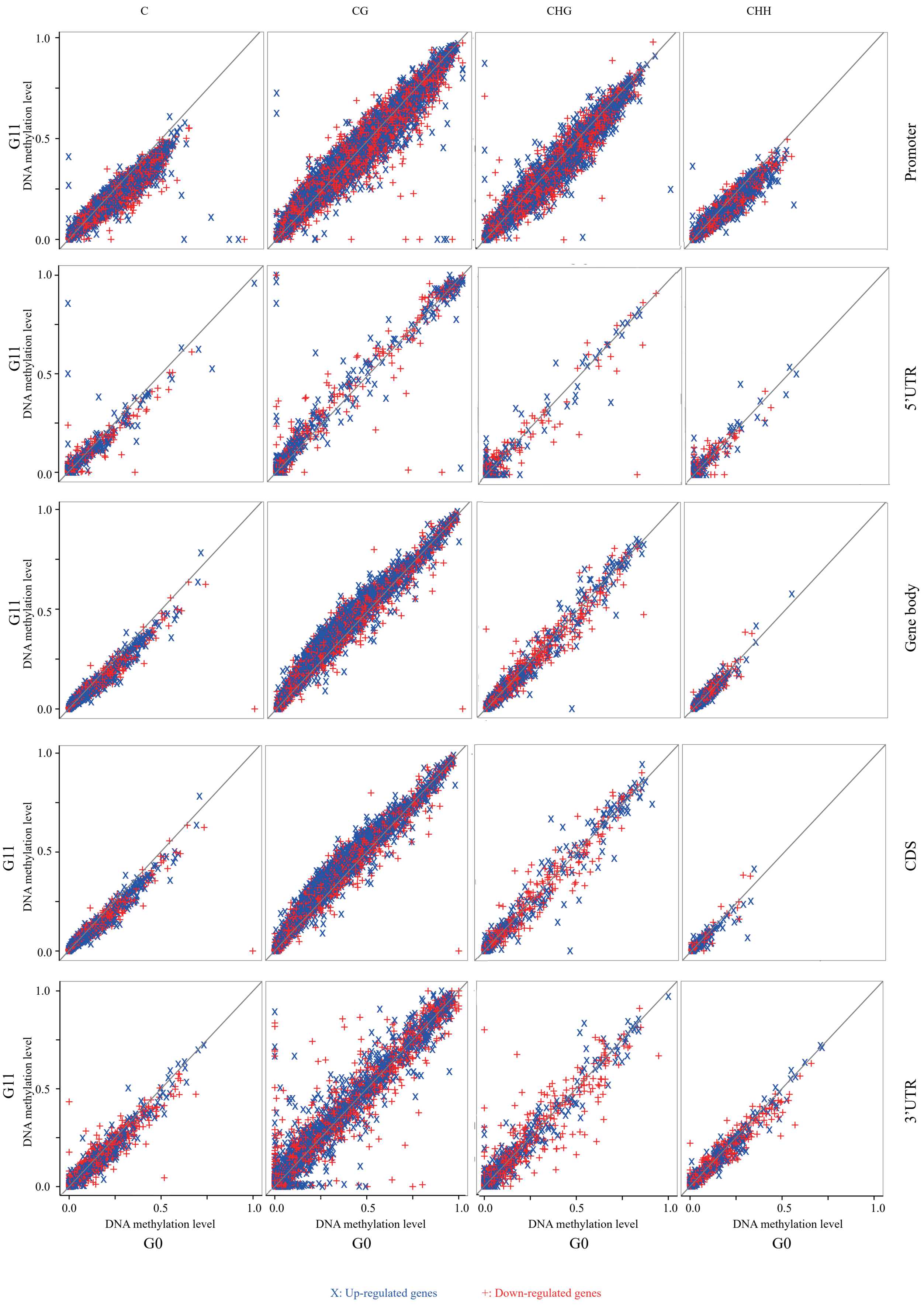
C
CG
CHG
CHH
Promoter
5'UTR
Gene body
CDS
3'UTR
G11
DNA methylation level
1.0
0.5
0.0
DNA methylation level
G0
X: Up-regulated genes
+: Down-regulated genes

Figure S7

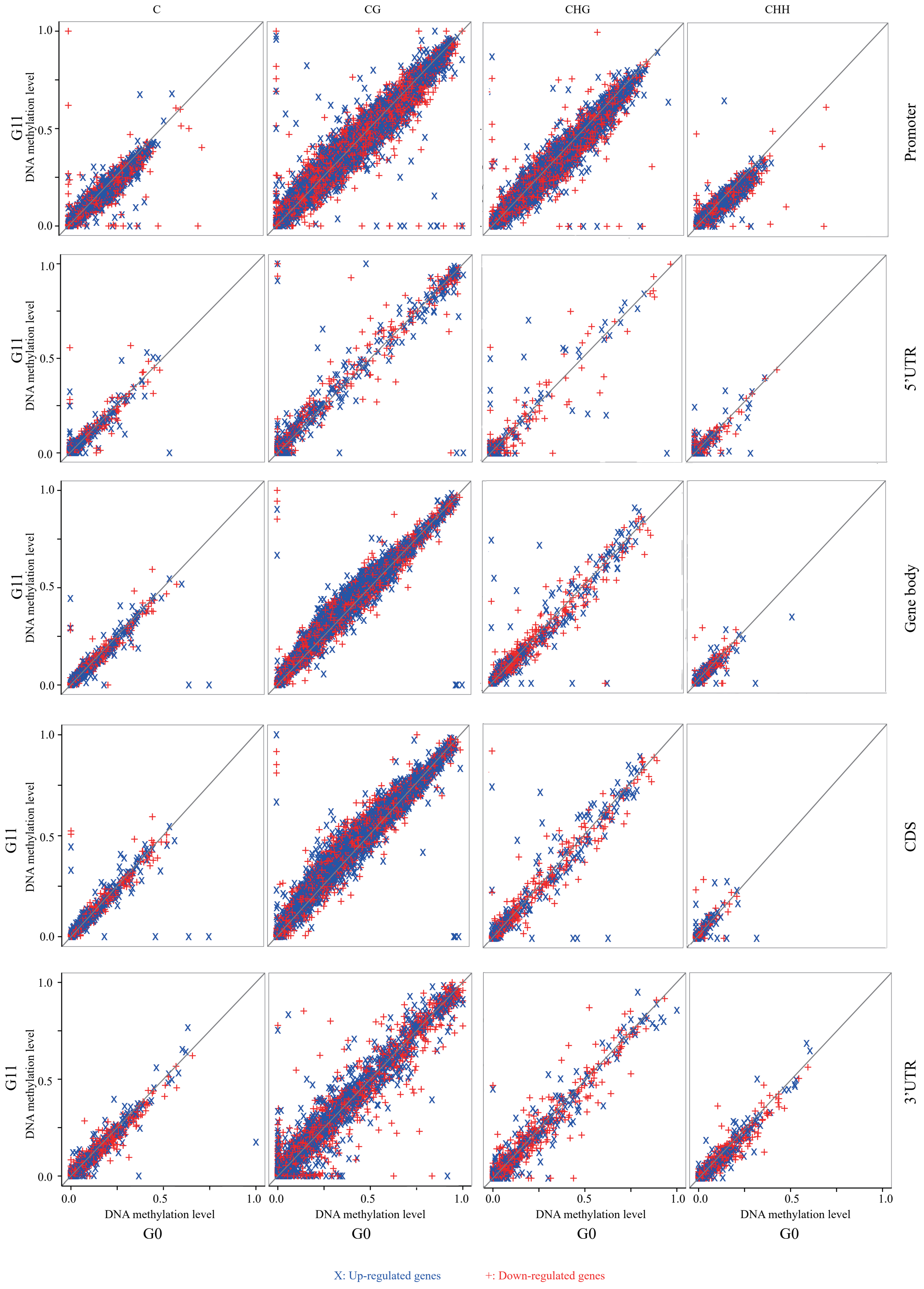

C
CG
CHG
CHH
Promoter
5'UTR
Gene body
CDS
3'UTR
G11
DNA methylation level
1.0
0.5
0.0
DNA methylation level
G0
X: Up-regulated genes
+: Down-regulated genes

Figure S8

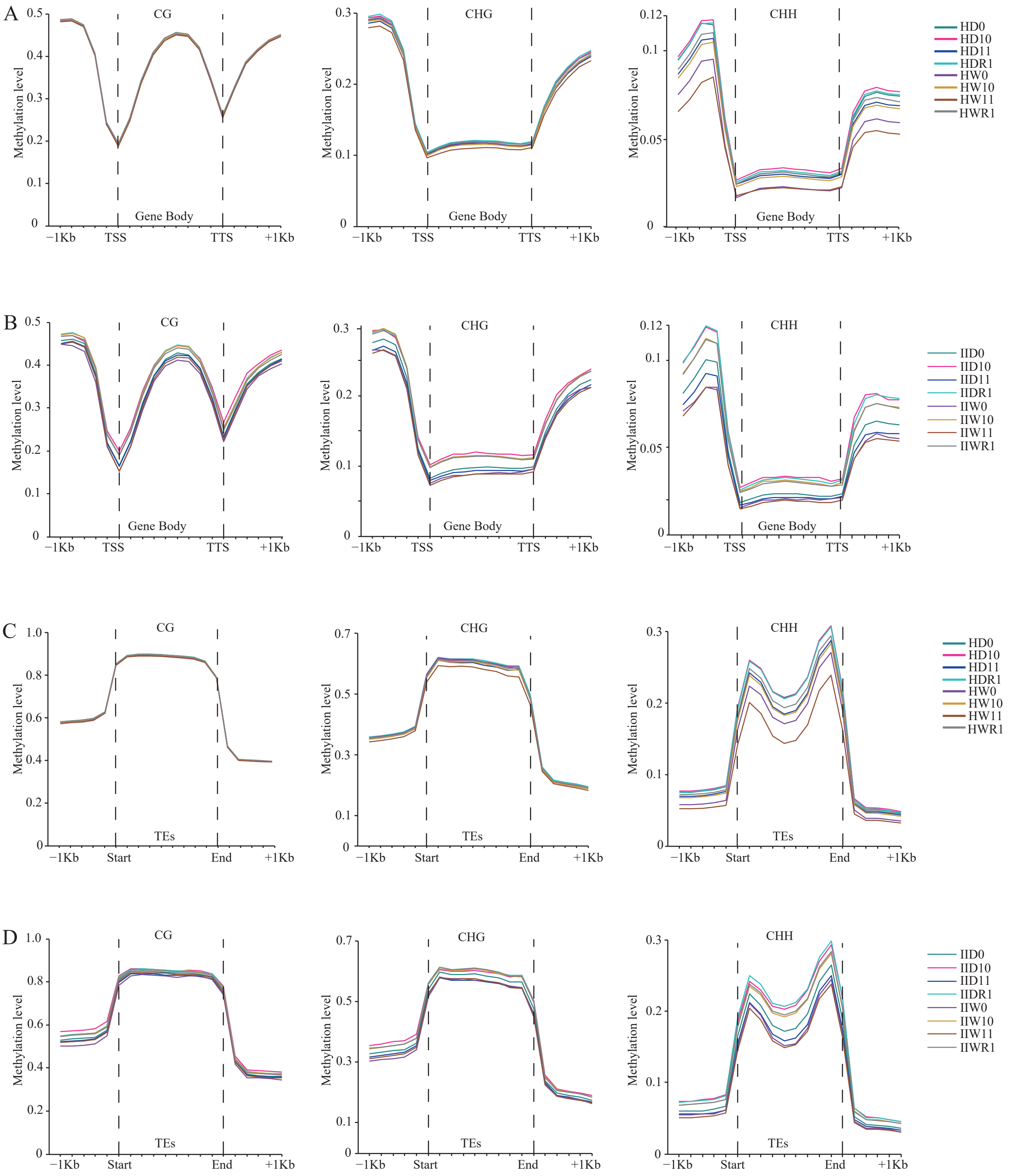

A
B
C
D
CG
CHG
CHH
Methylation level
Gene Body
TEs
−1Kb
TSS
TTS
+1Kb
Start
End
HD0
HD10
HD11
HDR1
HW0
HW10
HW11
HWR1

Figure S9

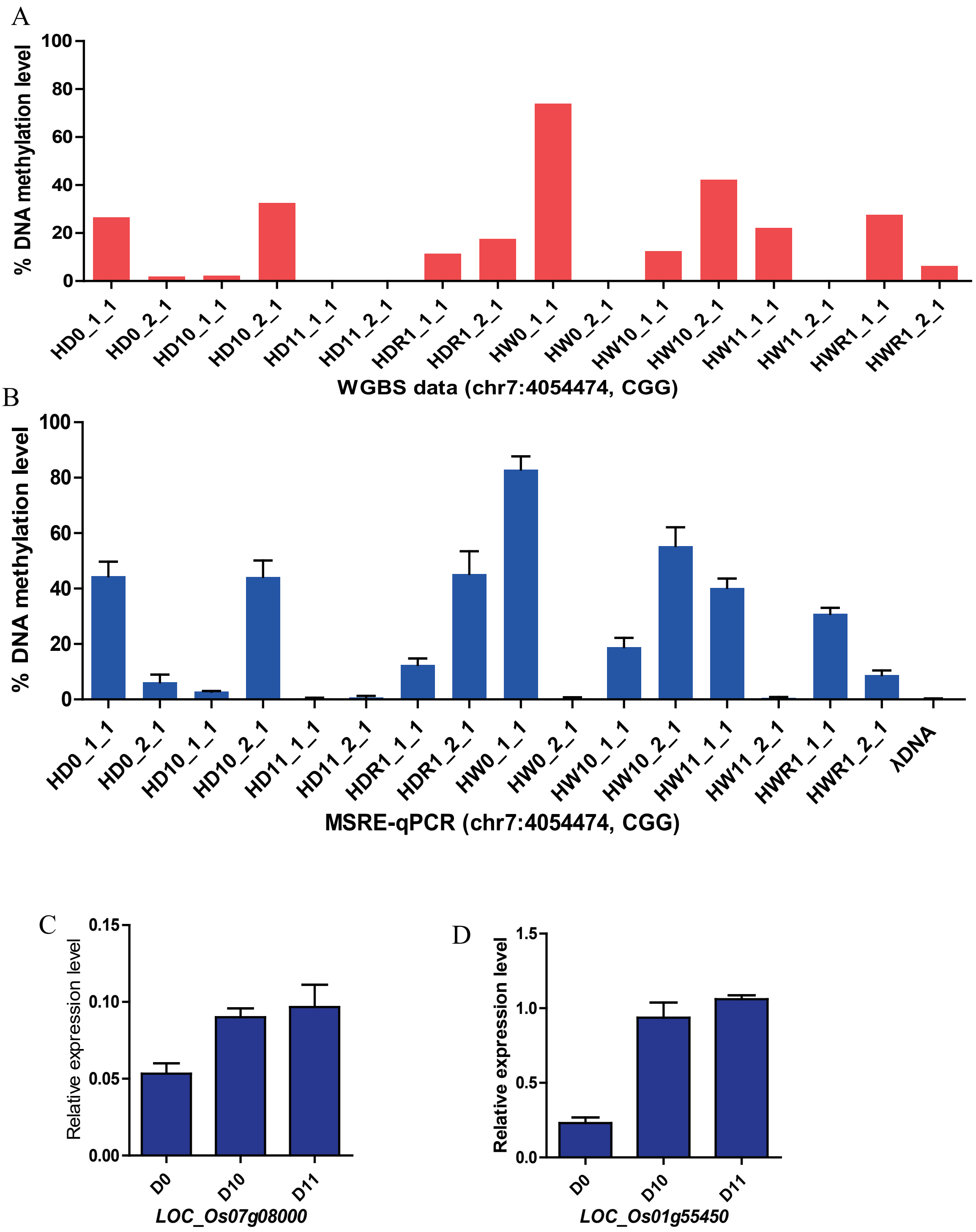
A
% DNA methylation level
100
80
60
40
20
0
HD0_1_1
HD0_2_1
HD10_1_1
HD10_2_1
HD11_1_1
HD11_2_1
HDR1_1_1
HDR1_2_1
HW0_1_1
HW0_2_1
HW10_1_1
HW10_2_1
HW11_1_1
HW11_2_1
HWR1_1_1
HWR1_2_1
WGBS data (chr7:4054474, CGG)
B
% DNA methylation level
100
80
60
40
20
0
HD0_1_1
HD0_2_1
HD10_1_1
HD10_2_1
HD11_1_1
HD11_2_1
HDR1_1_1
HDR1_2_1
HW0_1_1
HW0_2_1
HW10_1_1
HW10_2_1
HW11_1_1
HW11_2_1
HWR1_1_1
HWR1_2_1
λDNA
MSRE-qPCR (chr7:4054474, CGG)
C
Relative expression level
0.15
0.10
0.05
0.00
D0
D10
D11
LOC_Os07g08000
D
Relative expression level
1.5
1.0
0.5
0.0
D0
D10
D11
LOC_Os01g55450

Figure S10

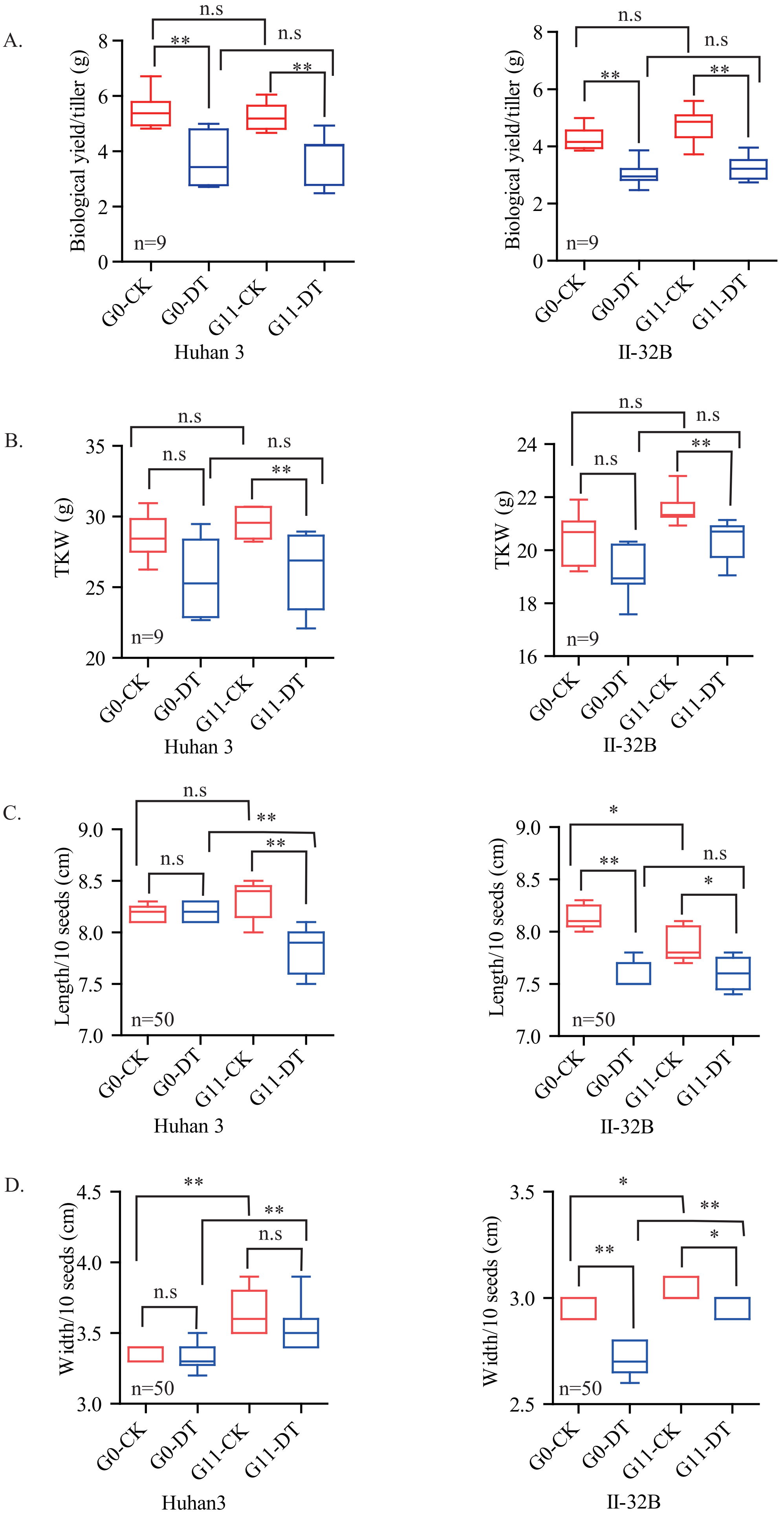
A.
Biological yield/tiller (g)
n=9
G0-CK
G0-DT
G11-CK
G11-DT
Huhan 3
II-32B
n.s
**
B.
TKW (g)
n=9
Huhan 3
II-32B
C.
Length/10 seeds (cm)
n=50
Huhan 3
II-32B
*
D.
Width/10 seeds (cm)
n=50
Huhan3
II-32B

Table S1 WGBS data set details

Varieties	Samples	Conversion rate	Total reads	Uniquely mapped reads	Genome coverage (×)	Strand_specifc coverage (×)	Genome coverage (%)	C coverage ⩾1				C coverage ⩾3				mC in C coverage ⩾3			
								all Cs	CG context	CHG context	CHH context	all Cs	CG context	CHG context	CHH context	all Cs	CG context	CHG context	CHH context
Huhan3	HD0_1	99.77%	181,843,462	125,143,714	30.13	14.50	82.78%	134,778,921	25,899,372	22,982,225	85,897,324	115,111,104	23,800,011	20,139,452	71,171,641	34,273,371	12,946,132	7,707,163	13,620,076
	HD0_2	99.66%	181,468,332	119,818,814	28.84	14.00	83.11%	135,317,909	25,722,220	23,307,202	86,288,487	116,917,291	23,126,381	20,897,055	72,893,855	39,998,137	13,029,061	9,125,405	17,843,671
	HD10_1	99.67%	170,237,156	113,494,818	27.32	12.80	85.24%	138,788,649	25,569,033	23,471,771	89,747,845	126,614,692	23,071,968	21,677,947	81,864,777	41,370,197	12,374,106	9,136,501	19,859,590
	HD10_2	99.50%	172,138,944	113,008,580	27.20	12.70	85.71%	139,543,495	25,855,466	23,640,339	90,047,690	127,923,774	23,595,091	21,973,035	82,355,648	44,859,744	13,119,581	9,819,964	21,920,199
	HD11_1	99.45%	181,404,200	122,665,634	29.53	13.30	85.71%	139,553,484	25,472,271	23,477,866	90,603,347	129,153,505	22,883,734	21,770,629	84,499,142	43,354,914	12,268,546	9,445,286	21,641,082
	HD11_2	99.64%	179,632,364	124,114,058	29.88	14.30	84.52%	137,609,429	25,779,960	23,490,412	88,339,057	122,009,730	23,278,838	21,402,092	77,328,800	40,738,165	12,994,102	9,237,930	18,506,133
	HDR1_1	99.71%	168,650,868	113,943,064	27.43	12.80	84.93%	138,288,631	25,449,417	23,419,072	89,420,142	125,248,874	22,898,996	21,541,077	80,808,801	39,855,075	12,134,554	8,920,872	18,799,649
	HDR1_2	99.62%	173,428,974	113,237,684	27.26	12.80	84.89%	138,208,809	25,679,505	23,510,180	89,019,124	123,811,778	23,200,280	21,538,810	79,072,688	42,235,255	12,761,812	9,395,241	20,078,202
	HW0_1	99.82%	181,572,724	124,068,854	29.87	14.20	82.75%	134,736,133	25,334,517	23,108,498	86,293,118	117,189,580	22,570,982	20,773,590	73,845,008	33,164,132	11,862,437	8,316,399	12,985,296
	HW0_2	99.82%	180,682,974	121,180,814	29.17	13.60	82.96%	135,080,424	25,315,094	23,101,029	86,664,301	119,184,083	22,766,117	20,973,695	75,444,271	34,992,436	11,942,820	8,255,356	14,794,260
	HW10_1	99.67%	171,885,916	114,115,948	27.47	12.70	85.28%	138,858,347	25,530,503	23,494,811	89,833,033	126,974,540	23,088,243	21,749,555	82,136,742	39,837,387	12,344,640	9,053,453	18,439,294
	HW10_2	99.62%	172,890,296	118,430,730	28.51	13.20	85.41%	139,068,088	25,574,443	23,500,871	89,992,774	127,846,154	23,210,920	21,841,251	82,793,983	41,496,929	12,485,486	9,244,921	19,766,522
	HW11_1	99.52%	181,721,480	122,517,846	29.49	13.40	85.93%	139,916,463	25,688,171	23,591,987	90,636,305	129,508,439	23,237,654	21,948,067	84,322,718	41,090,608	12,586,808	9,514,964	18,988,836
	HW11_2	99.60%	188,305,712	133,031,848	32.02	14.20	84.68%	137,881,707	24,809,467	23,081,118	89,991,122	126,674,229	21,791,232	21,118,272	83,764,725	37,033,178	10,921,689	8,436,745	17,674,744
	HWR1_1	99.63%	172,559,304	120,799,374	29.08	13.50	85.38%	139,010,122	25,540,229	23,485,490	89,984,403	127,795,790	23,143,137	21,814,259	82,838,394	41,143,682	12,427,818	9,186,111	19,529,753
	HWR1_2	99.57%	172,407,024	116,059,152	27.94	13.20	85.00%	138,400,891	25,695,306	23,507,118	89,198,467	124,674,764	23,243,049	21,612,012	79,819,703	43,276,262	12,845,440	9,565,955	20,864,867
	Average	99.64%	176,926,858	119,726,933	28.82	13.45	84.64%	137,815,094	25,557,186	23,385,624	88,872,284	124,164,895	23,056,665	21,423,175	79,685,056	39,919,967	12,440,315	9,022,642	18,457,011
II-32B	IID0_1	99.81%	179,208,446	105,882,346	25.49	11.80	76.53%	124,606,752	22,505,691	21,139,157	80,961,904	109,445,346	19,539,635	18,877,570	71,028,141	17,841,104	8,470,598	4,887,965	4,482,541
	IID0_2	99.69%	180,575,660	110,123,640	26.51	11.74	74.94%	122,009,797	21,193,500	20,426,655	80,389,642	108,786,140	18,142,948	18,259,556	72,383,636	15,564,298	7,179,025	4,214,566	4,170,707
	IID10_1	99.62%	169,465,964	92,320,586	22.22	10.50	77.16%	125,636,593	23,277,277	21,514,826	80,844,490	107,736,917	20,247,067	18,968,368	68,521,482	20,876,730	9,444,766	5,496,374	5,935,590
	IID10_2	99.64%	172,976,524	97,642,944	23.51	10.90	78.07%	127,104,856	23,215,330	21,577,980	82,311,546	113,591,799	20,516,542	19,540,814	73,534,443	20,416,049	9,379,039	5,464,726	5,572,284
	IID11_1	99.76%	182,207,216	107,955,586	25.99	11.51	75.78%	123,386,051	21,711,165	20,683,463	80,991,423	110,412,607	18,606,111	18,508,751	73,297,745	15,713,605	7,425,237	4,173,869	4,114,499
	IID11_2	99.77%	181,985,458	111,351,458	26.81	11.95	76.09%	123,895,989	21,810,806	20,810,387	81,274,796	111,292,666	18,948,666	18,769,114	73,574,886	16,788,116	7,934,134	4,655,830	4,198,152
	IIDR1_1	99.61%	172,691,624	95,014,666	22.87	10.82	77.03%	125,412,951	23,270,446	21,471,535	80,670,970	108,402,621	20,415,788	19,075,047	68,911,786	20,939,962	9,550,942	5,548,945	5,840,075
	IIDR1_2	99.67%	168,906,748	97,082,758	23.37	10.68	77.09%	125,509,909	22,733,330	21,291,960	81,484,619	110,987,576	19,796,498	19,095,434	72,095,644	18,892,254	8,809,127	5,087,296	4,995,831
	IIW0_1	99.77%	174,162,830	98,559,862	23.73	10.65	75.72%	123,284,376	21,753,883	20,702,762	80,827,731	109,820,871	18,639,055	18,487,462	72,694,354	15,020,086	7,394,301	4,168,991	3,456,794
	IIW0_2	99.67%	179,175,868	101,958,348	24.54	10.99	75.25%	122,521,345	21,456,060	20,545,049	80,520,236	109,237,853	18,340,989	18,364,934	72,531,930	15,005,787	7,331,058	4,147,947	3,526,782
	IIW10_1	99.67%	172,150,684	94,517,288	22.75	10.65	76.32%	124,265,188	23,133,611	21,355,621	79,775,956	106,058,129	20,132,600	18,806,685	67,118,844	20,037,173	9,378,915	5,409,560	5,248,698
	IIW10_2	99.59%	171,356,848	100,206,278	24.12	11.22	77.76%	126,614,283	23,118,247	21,481,104	82,014,932	113,139,576	20,399,930	19,447,415	73,292,231	19,520,340	9,206,569	5,293,457	5,020,314
	IIW11_1	99.76%	181,109,402	110,061,804	26.49	11.92	74.35%	121,055,079	20,998,835	20,268,403	79,787,841	107,194,766	17,892,937	18,028,734	71,273,095	13,736,136	6,800,755	3,832,241	3,103,140
	IIW11_2	99.69%	180,466,108	110,114,766	26.51	11.89	75.34%	122,670,475	21,408,548	20,548,784	80,713,143	109,081,449	18,250,866	18,315,750	72,514,833	15,021,261	7,211,970	4,095,376	3,713,915
	IIWR1_1	99.66%	173,564,342	99,746,186	24.01	11.15	77.67%	126,467,312	23,175,612	21,509,377	81,782,323	112,089,036	20,432,617	19,396,654	72,259,765	19,428,749	9,250,103	5,308,347	4,870,299
	IIWR1_2	99.66%	173,772,026	98,526,050	23.72	11.06	77.68%	126,482,560	23,228,177	21,521,367	81,733,016	112,391,564	20,581,770	19,459,748	72,350,046	20,437,269	9,375,422	5,466,639	5,595,208
	Average	99.69%	175,860,984	101,941,535	24.54	11.21	76.42%	124,432,720	22,374,407	21,053,027	81,005,286	109,979,307	19,430,251	18,837,627	71,711,429	17,827,432	8,383,873	4,828,258	4,615,302

Reference genome：
Nipponbare
all Cs: 162,817,618
CG context: 30,862,860
CHG context: 27,417,182

Table S2 The numbers of DMPs, SMPs and DMRs between treated and untreated plants and between generations.

Combinations	C-DMPs	CG-DMPs	CHG-DMPs	CHH-DMPs	C-SMPs	CG-SMPs	CHG-SMPs	CHH-SMPs	DMRs	Hyper-DMRs	Hypo-DMRs
**Huhan3**											
HD10-HD0	275,016	81,283	115,361	78,372	2,024,303	198,242	246,043	1,580,018	3,489	1,708	1,781
HD11-HD0	312,695	92,477	127,987	92,231	2,008,396	225,427	252,101	1,530,868	5,367	1,991	3,376
HD11-HD10	275,374	78,596	120,523	76,255	2,202,100	201,124	279,361	1,721,615	4,611	1,584	3,027
HD0-HW0	322,866	73,951	112,246	136,669	1,584,407	152,184	175,084	1,257,139	1,986	672	1,314
HD10-HW10	298,845	65,887	113,195	119,763	2,046,274	157,018	245,769	1,643,487	2,503	999	1,504
HD11-HW11	409,786	68,092	108,789	232,905	2,371,863	174,394	288,793	1,908,676	3,910	1,154	2,756
HD10R1-HW10R1	248,464	56,733	98,091	93,640	1,994,083	154,097	236,645	1,603,341	1,324	725	599
HW10-HW0	293,682	83,349	128,254	82,079	1,887,194	156,440	211,855	1,518,899	4,078	2,199	1,879
HW11-HW0	338,144	80,269	120,214	137,661	2,074,259	169,124	265,173	1,639,962	6,926	2,427	4,499
HW11-HW10	396,872	77,897	114,000	204,975	2,288,495	151,785	265,335	1,871,375	5,564	1,286	4,278
HD10R1-HD0	281,419	84,683	122,595	74,141	1,863,519	192,742	214,043	1,456,734	4,099	2,155	1,944
HD10R1-HD10	260,539	68,637	119,423	72,479	1,999,616	164,875	240,107	1,594,634	2,938	1,655	1,283
HW10R1-HW0	302,439	81,100	125,408	95,931	2,096,919	178,751	242,314	1,675,854	4,304	2,781	1,523
HW10R1-HW10	237,906	61,542	107,663	68,701	2,035,334	162,881	245,639	1,626,814	2,311	1,540	771
**II-32B**											
IID10-IID0	309,254	89,171	105,823	114,260	1,576,980	150,887	195,081	1,231,012	8,060	6,626	1,433
IID11-IID0	213,251	71,145	86,271	55,835	1,304,271	113,151	163,446	1,027,674	6,624	3,496	3,127
IID11-IID10	380,567	72,111	102,073	206,383	1,700,870	119,805	198,623	1,382,442	10,395	786	9,608
IID0-IIW0	199,616	58,075	72,054	69,487	1,335,324	85,853	166,043	1,083,428	2,179	629	1,549
IID10-IIW10	196,088	47,780	69,448	78,860	1,536,605	115,474	194,345	1,226,786	585	117	467
IID11-IIW11	215,972	80,277	75,212	60,483	1,352,897	83,186	175,008	1,094,703	1,997	878	1,118
IID10R1-IIW10R1	253,408	92,532	88,311	72,565	1,461,654	112,590	184,713	1,164,351	1,662	430	1,231
IIW10-IIW0	345,454	87,140	105,524	152,790	1,817,883	158,636	223,421	1,435,826	11,193	9,690	1,502
IIW11-IIW0	170,742	62,407	75,130	33,205	1,373,081	116,621	189,489	1,066,971	6,193	3,632	2,560
IIW11-IIW10	313,180	69,639	96,680	146,861	1,856,569	135,911	230,942	1,489,716	8,524	971	7,552
IID10R1_IID0	262,263	77,199	89,434	95,630	1,505,328	141,469	188,236	1,175,623	6,958	5,390	1,567
IID10R1_IID10	197,811	55,838	89,181	52,792	1,531,962	139,049	197,635	1,195,278	2,187	858	1,328
IIW10R1_IIW0	338,570	78,657	99,510	160,403	1,713,237	136,018	205,133	1,372,086	10,326	8,532	1,793
IIW10R1_IIW10	194,938	57,843	87,555	49,540	1,505,185	132,014	195,164	1,178,007	2,459	839	1,619

Table S3. Amount of non-TE genes which related to epimutations whose recurrent frequencies ⩾3 involved in seven GOSlim terms.

**GOSlim Accessions/Terms**	a	b	c	d	e	f	g	h
Epimutations with recurrent frequencies⩾3	480	3,467	186	1,858	3,260	10,908	2,058	6,594
GO:0009991/response to extracellular stimulus	5	1	1	2	7	6	2	6
GO:0009719/response to endogenous stimulus	5	11	1	30	19	59	11	25
GO:0009628/response to abiotic stimulus	9	21	5	30	31	76	18	48
GO:0009607/response to biotic stimulus	3	14	2	11	16	49	8	29
GO:0009605/response to external stimulus	0	2	0	4	2	10	0	3
GO:0006950/response to stress	14	38	7	49	57	125	33	79
GO:0040029/regulation of gene expression, epigenetic	1	1	1	3	7	6	1	8
Total (each gene was counted only once)	19	59	9	84	83	208	53	121
Total genes related to these epimutations	99	407	48	553	498	1,481	346	823
Proportions	19.19%	14.50%	18.75%	15.19%	16.67%	14.04%	15.32%	14.70%

Amount of genes related to hypo-methylated DMPs (a) and hyper-methylated DMPs (b) in Huhan3

Amount of genes related to hypo-methylated DMPs (c) and hyper-methylated DMPs (d) in II-32B

Amount of genes related to de-methylated SMPs (e) and re-methylated SMPs (f) in Huhan3

Amount of genes related to de-methylated SMPs (g) and re-methylated SMPs (h) in II-32B.

Table S4. Amount of non-TE genes that were related to transgenerational accumulated epimutations involved in seven GOSlim terms.

GOSlim Accessions/Terms	a	b	c	d	e	f	g	h
Transgenerational accumulated epimutations	7,535	18,279	3,547	19,666	178,442	122,488	173,739	72,121
GO:0009991/response to extracellular stimulus	0	2	1	6	8	2	6	1
GO:0009719/response to endogenous stimulus	3	12	1	13	20	6	35	4
GO:0009628/response to abiotic stimulus	4	22	2	30	45	5	40	6
GO:0009607/response to biotic stimulus	1	15	2	16	25	4	29	2
GO:0009605/response to external stimulus	0	2	0	2	8	0	3	0
GO:0006950/response to stress	8	52	5	67	64	20	65	10
GO:0040029/regulation of gene expression, epigenetic	0	1	0	4	2	1	15	1
Total (each gene was counted only once)	11	66	7	90	90	27	119	13
Total genes related to transgenerational epimutations*	61	377	45	589	434	102	571	62
Proportions	18.03%	17.51%	15.56%	15.28%	20.74%	26.47%	20.84%	20.97%

Amount of genes related to hypo-methylated DMPs (a) and hyper-methylated DMPs (b) in Huhan3

Amount of genes related to hypo-methylated DMPs (c) and hyper-methylated DMPs (d) in II-32B

Amount of genes related to de-methylated SMPs (e) and re-methylated SMPs (f) in Huhan3

Amount of genes related to de-methylated SMPs (g) and re-methylated SMPs (h) in II-32B.

*Non-TE genes that contained at least 3 transgenerational DMPs or at least 10 transgenerational SMPs

Table S5 Amount of functional genes that overlapped with drought-induced recurrent DMRs and transgenerational accumulated DMRs in Huhan3 and II-32B.

	Drought-induced recurrent DMRs[1)]				Transgenerational accumulated DMRs			
	Huhan3		II-32B		Huhan3		II-32B	
	Hypo-methylated	Hyper-methylated	Hypo-methylated	Hyper-methylated	Hypo-methylated	Hyper-methylated	Hypo-methylated	Hyper-methylated
Total DMRs	345	250	262	55	462	515	514	1,460
DMRs that related to genes	107	85	105	18	162	202	180	536
Percentages of these DMRs (%)	31.01	34	40.08	32.73	35.06	39.22	35.02	36.71
Genes (DMRs in promoter)	66	54	40	12	112	114	120	294
Genes (DMRs in body)	49	42	78	7	77	137	87	325
Total genes[2)]	110	89	110	19	170	217	194	572
Stress responsive genes[3)]	14	14	21	1	18	26	21	44
Percentages of these genes (%)	12.73	15.73	19.09	5.26	10.59	11.98	10.82	7.69
References (figures and supplemental tables)	Figure S3A Table S8A	Figure S3A Table S8B	Figure S3B Table S8C	Figure S3B Table S8D	Figure 6A Table S8E	Figure 6A Table S8F	Figure 6B Table S8G	Figure 6B Table S8H

[1)] DMRs that recurrent frequencies ≥ 2 in comparisons between drought-treated and well-watered plants of four generations.

[2)] Some genes overlapped with DMRs in both their promoters and bodies, and each gene was counted only once.

[3)] Genes that directly participated in stress response pathways.

Table S6 mRNA-seq data set details

Sample	Total_reads	Mapped_reads	Uniq_reads	Mapped_rate	Uniq_rate
HD0_1	36,901,082	33,016,422	26,397,060	0.8947	0.7153
HD0_2	38,102,824	33,401,600	25,806,642	0.8766	0.6773
HD10_1	36,971,644	33,295,268	26,581,604	0.9006	0.719
HD10_2	38,632,108	33,649,300	27,953,416	0.871	0.7236
HD11_1	39,507,974	34,934,156	27,868,168	0.8842	0.7054
HD11_2	43,441,314	39,895,862	32,871,528	0.9184	0.7567
HDR1_1	34,533,124	32,026,276	24,846,110	0.9274	0.7195
HDR1_2	38,845,490	35,232,488	28,564,600	0.907	0.7353

Table S7 Seven experimental array-combinations

Experiment	Control1	Control2	Control3	Treat1	Treat2	Treat3	GSE	Annotation
Drought, Azucena leaf	GSM591761	GSM591762	GSM591763	GSM591767	GSM591768	GSM591769	GSE24048	Expression data from field droughted rice plants
Drought, Bala leaf	GSM591764	GSM591765	GSM591766	GSM591770	GSM591771	GSM591772	GSE24048	Expression data from field droughted rice plants
Drought, IR64	E_MEXP_2401_INA	E_MEXP_2401_INB	E_MEXP_2401_INC	E_MEXP_2401_ISA	E_MEXP_2401_ISB	E_MEXP_2401_ISC	E-MEXP-2401	Transcription profiling of Oryza sativa subtypes Cultivar Nagina-22 (N22) and IR64 subtypes under normal and drought conditions
Drought, N22	E_MEXP_2401_NNA	E_MEXP_2401_NNB	E_MEXP_2401_NNC	E_MEXP_2401_NSA	E_MEXP_2401_NSB	E_MEXP_2401_NSC	E-MEXP-2401	Transcription profiling of Oryza sativa subtypes Cultivar Nagina-22 (N22) and IR64 subtypes under normal and drought conditions
Drought, leaves at tillering stage	GSM645322	GSM645323	GSM645324	GSM645325	GSM645326	GSM645327	GSE26280	Genome-wide temporal-spatial gene expression profiling of drought responsiveness in rice
Drought, leaves at panicle elongation stage	GSM645334	GSM645335	GSM645336	GSM645337	GSM645338	GSM645339	GSE26280	Genome-wide temporal-spatial gene expression profiling of drought responsiveness in rice
Drought, leaves at booting stage	GSM645346	GSM645347	GSM645348	GSM645349	GSM645350	GSM645351	GSE26280	Genome-wide temporal-spatial gene expression profiling of drought responsiveness in rice
